# Where does diversity come from? Linking geographical patterns of morphological, genetic, and environmental variation in wall lizards

**DOI:** 10.1186/s12862-018-1237-7

**Published:** 2018-08-22

**Authors:** Antigoni Kaliontzopoulou, Catarina Pinho, Fernando Martínez-Freiría

**Affiliations:** CIBIO/InBIO Research Centre in Biodiversity and Genetic Resources, University of Porto, Campus de Vairão, Rua Padre Armando Quintas, N° 7.4485-661 Vairão, Vila do Conde, Portugal

**Keywords:** Individuals, Population divergence, Generalized dissimilarity modelling, Geometric morphometrics

## Abstract

**Background:**

Understanding how phenotypic variation scales from individuals, through populations, up to species, and how it relates to genetic and environmental factors, is essential for deciphering the evolutionary mechanisms that drive biodiversity. We used two species of *Podarcis* wall lizards to test whether phenotypic diversity within and divergence across populations follow concordant patterns, and to examine how phenotypic variation responds to genetic and environmental variability across different hierarchical levels of biological organization, in an explicit geographic framework.

**Results:**

We found a general concordance of phenotypic variation across hierarchical levels (i.e. individuals and populations). However, we also found that within-population diversity does not exhibit a coherent geographic structure for most traits, while among-population divergence does, suggesting that different mechanisms may underlie the generation of diversity at these two levels. Furthermore, the association of phenotypic variation with genetic and environmental factors varied extensively between hierarchical levels and across traits, hampering the identification of simple rules to explain what yields diversity.

**Conclusions:**

Our results in some cases comply with general ecological and evolutionary predictions, but in others they are difficult to explain in the geographic framework used, suggesting that habitat characteristics and other regulatory mechanisms may have a more substantial contribution in shaping phenotypic diversity.

**Electronic supplementary material:**

The online version of this article (10.1186/s12862-018-1237-7) contains supplementary material, which is available to authorized users.

## Background

Variation is the cornerstone of evolution. It is the raw material for the action of selection and a central component for identifying how phenotypic diversification occurs across all levels of biological organization [1]. The unit of phenotypic variation is the individual, through which fitness is optimized via effects on survival and reproduction. Selective influences and stochastic processes like drift accumulate across individuals, yielding variation at the population level [2]. When intraspecific differentiation is further reinforced through mechanisms of population divergence, reproductive isolation may gradually develop, which produces phenotypic variation among different species [3]. Through this pipeline across levels of biological organization, we may conceptually trace variation from individuals, through populations of the same species, to macroevolutionary diversification across species. Due to its relevance for understanding evolution, phenotypic diversity has been explored at all these hierarchical levels. Comparative studies of phenotypic evolution combine species phenotypic data and phylogenetic trees, to explore, for instance, how shared ancestry and biological traits determine macroevolutionary diversification [4]. Similar questions have been addressed, using a different set of analytical tools, to examine phenotypic variation at the intraspecific level from ecological, evolutionary, and conservation-focused perspectives, as it could determine the potential of a species to adapt to local conditions and variations of these across time (e.g. [5, 6]).

Common to all these approaches is the recognition of the importance of both genetic and environmental factors in shaping phenotypic variation. The connection between genotype and phenotype is a central topic in evolutionary biology, and in recent years our understanding of the genetic architecture underlying many phenotypic traits has been highly enhanced through the application of modern sequencing techniques and quantitative genetic tools [7]. Most usually, however, indirect associations are established between genetic and phenotypic patterns, by using neutral markers to characterize genetic variation. In this type of studies, the hypothesis tested is usually that of a positive correlation between genetic and phenotypic variation [8]. At the intraspecific level, greater degrees of (neutral) genetic divergence between populations are typically associated to lower connectivity between them, and the more differentiated these populations are predicted to be phenotypically, *under the null hypothesis of random accumulation of variation*. Of course, evolution, and particularly that of the phenotype, rarely occurs under stochastic processes alone. Instead, phenotypic traits are usually also correlated with the environment where organisms live, through effects on physiological, functional and ecological performance [9]. Across populations, local adaptation and phenotypic plasticity are major frameworks for studying associations between the phenotype and the environment, and for elucidating the mechanisms underlying intraspecific phenotypic diversity [10, 11]. As the phenotypic optima are expected to be similar for individuals of the same species that inhabit similar environments, we frequently observe population-level responses to environmental variation, and therefore it is not unusual for patterns of phenotypic divergence to correlate to environmental differences across populations [5, 12].

In all these cases, additional perspectives may be gained by considering the geographic structure of variation. Because both genetic and environmental variation are frequently geographically structured, phenotypic traits also tend to exhibit some geographic pattern (e.g. [10]). Genetic variation in particular may exhibit geographic structuring due to the spatial dynamics of migration and population differentiation [13, 14], or as a result of direct environmental influences, as described by the isolation-by-distance family of models [15]. In a similar fashion, environmental characteristics exhibit strong spatial autocorrelation at certain geographical scales, following diffusion mathematical models (e.g. [16]). Similar modelling techniques have been frequently used to examine the spatial structure of phenotypic variation by focusing on differentiation across populations [17–20]. A less appreciated aspect is that the degree of phenotypic variation *within* populations could also exhibit a geographic structure, determined by underlying links with genetic and environmental traits. This geographic structuring of both within-population *diversity* and among-population *divergence* needs to be statistically taken into account when trying to establish links between phenotypic and genetic or environmental factors. On the other hand, it also provides the opportunity to examine patterns of variation in a geographically explicit framework [21], which can aid in pinpointing patterns of variation that may remain unaccounted for by the genetic and environmental factors explicitly considered in the examined models.

Here, we investigated how phenotypic variation emerging from individuals is expressed as diversity within and divergence across populations of the same species, and at linking morphological, genetic, and environmental patterns in a geographically explicit analytical framework. For this purpose, we sampled morphological, genetic and environmental variation across populations of two species of wall lizards of the genus *Podarcis*. These lizards are a particularly intriguing system for exploring the processes involved in phenotypic differentiation, as they exhibit high intraspecific morphological variation, but a relatively conserved morphology across species, which results in high levels of cryptic genetic diversity [22, 23]. Using exactly the same set of individual specimens, we quantified a series of morphological traits and used genetic sampling to characterize recent population connectivity and diversity, and evolutionary history. Based on these individually-matched samples, we addressed the following questions: 1) Are patterns of morphological variation concordant at the within- and among-population levels? This is expected if phenotypic diversity responds to the same evolutionary influences – either stochastic or adaptive – at the individual and population levels. 2) Is diversity within populations and divergence among them geographically structured? Here, geographic structure, particularly if common across scales and traits, would point to direct, causal links between phenotypic, genetic, and environmental variation. 3) Which is the relative contribution of genetic and environmental factors in shaping morphological variation within and across populations? If variation in phenotypic traits is more strongly associated to population dynamics and evolutionary history, this will point to drift as a major factor shaping diversity patterns; alternatively, if it is strongly influenced by environmental factors, this will reflect plastic and adaptive mechanisms. By addressing these questions, we aimed at providing new insights into how phenotypic variation scales up from individuals to populations of the same species, and to examine whether it responds to the same genetic and environmental influences across different hierarchical levels of biological organization.

## Results

### Patterns of morphological variation

**Fig. 1:**
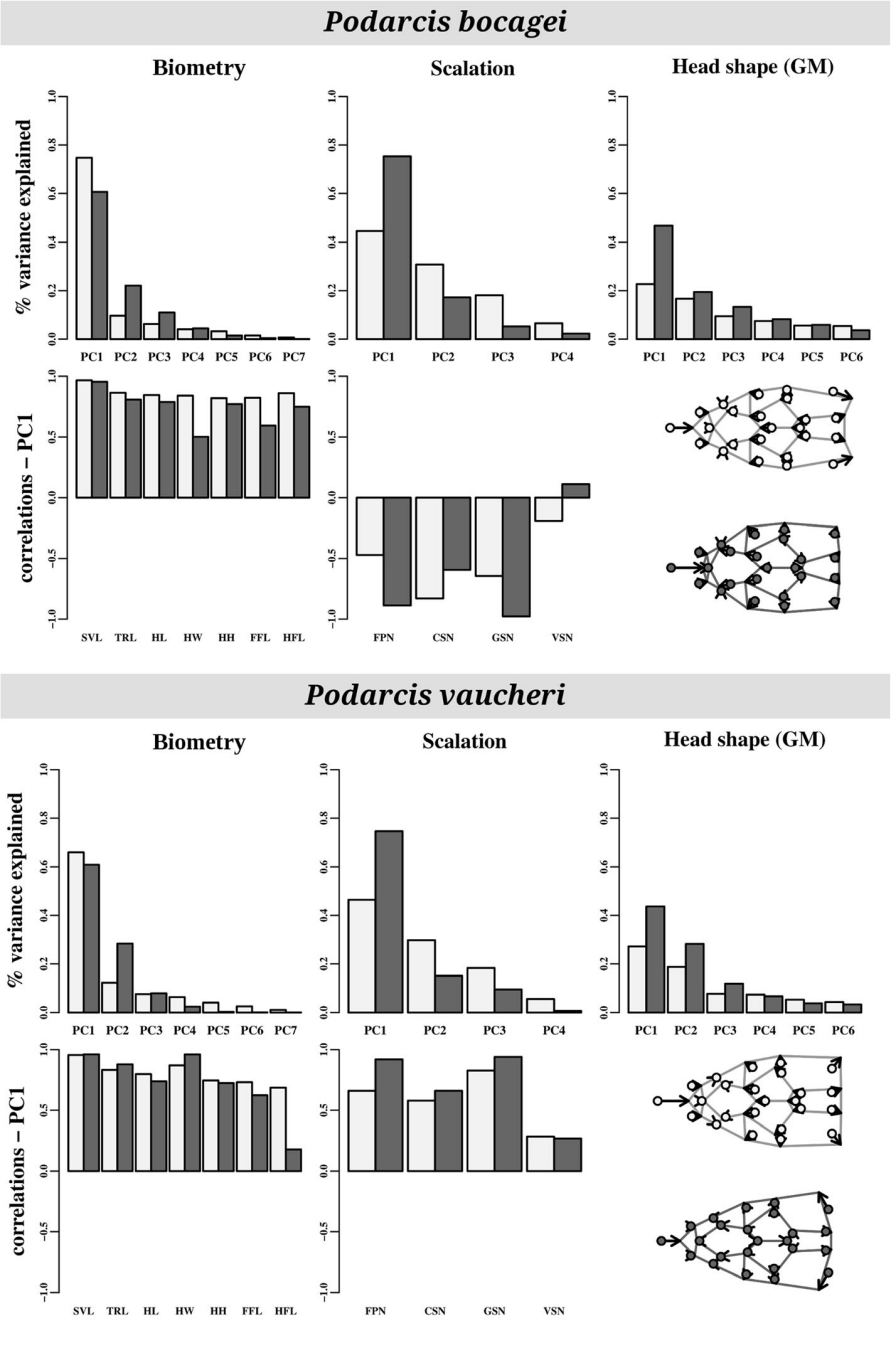
General structure of morphological variation in each species as visualized through principal components analysis of all individuals (light grey) or population means (dark grey) based on the multivariate set of biometric and scalation traits, and geometric morphometric data used to quantify head shape. Top: percentage of variance explained by each principal component. Bottom: correlations between the first PC and raw variables for each set of traits. For head shape as quantified through geometric morphometrics, deformation grids illustrate shape variation from the minimum to the maximum extreme of the first PC. See methods for variable abbreviations

The examination of the principal components (PC) of morphological variation across individuals and across populations indicated that, although the amount of variance captured differed between the two analyses, variation patterns were transversal to both hierarchical levels for all traits, except for head shape, in both species. Indeed, the structure of phenotypic variation as expressed by PC eigenvectors of the individual-based and population-based analyses did not differ significantly for neither linear biometric traits (*P. bocagei*: θ = 13.8°, *p* = 0.918; *P. vaucheri*: θ = 15.0°, *p* = 0.125), nor scalation (*P. bocagei*: θ = 32.2°, *p* = 0.155; *P. vaucheri*: θ = 14.6°, *p* = 0.286). The main direction of biometric variation was related to body size, both at individual and population analyses, as indicated by high, equal-sign loadings of all biometric traits on PC1 in both species (Fig. 1). This also resulted in a high eigenvalue for the first PC in both analyses and species, with more than 60% of variance explained. For scalation traits, the first PC generally correlated to higher scale counts for all examined traits, with a lower relative contribution of VSN, again under a common pattern across levels (Fig. 1). Instead, for head shape as quantified using geometric morphometrics, the direction of principal component eigenvectors differed significantly across individuals vs. across populations for both species (*P. bocagei*: θ = 50.7°, *p* = 0.001; *P. vaucheri*: θ = 50.4°, *p* = 0.001): while in both cases the first PC was related to a relative shortening of the snout, individual variation was also associated to a widening of the posterior region of the head, whereas population differentiation was rather related to a reduction of this area (Fig. 1).

### Geographic structure

Although interpolations were mostly accurate (Additional file 1: Table A8.1), within-population diversity values for studied traits did not exhibit a geographic structure, with the exception of body size in *P. bocagei* (Additional file 1: Figure A8). Spatial variation in interpolated traits was inexistent or extremely reduced and frequently presented spatial artefacts (e.g. body size in *P. vaucheri*; Additional file 1: Figure A8).

**Fig. 2:**
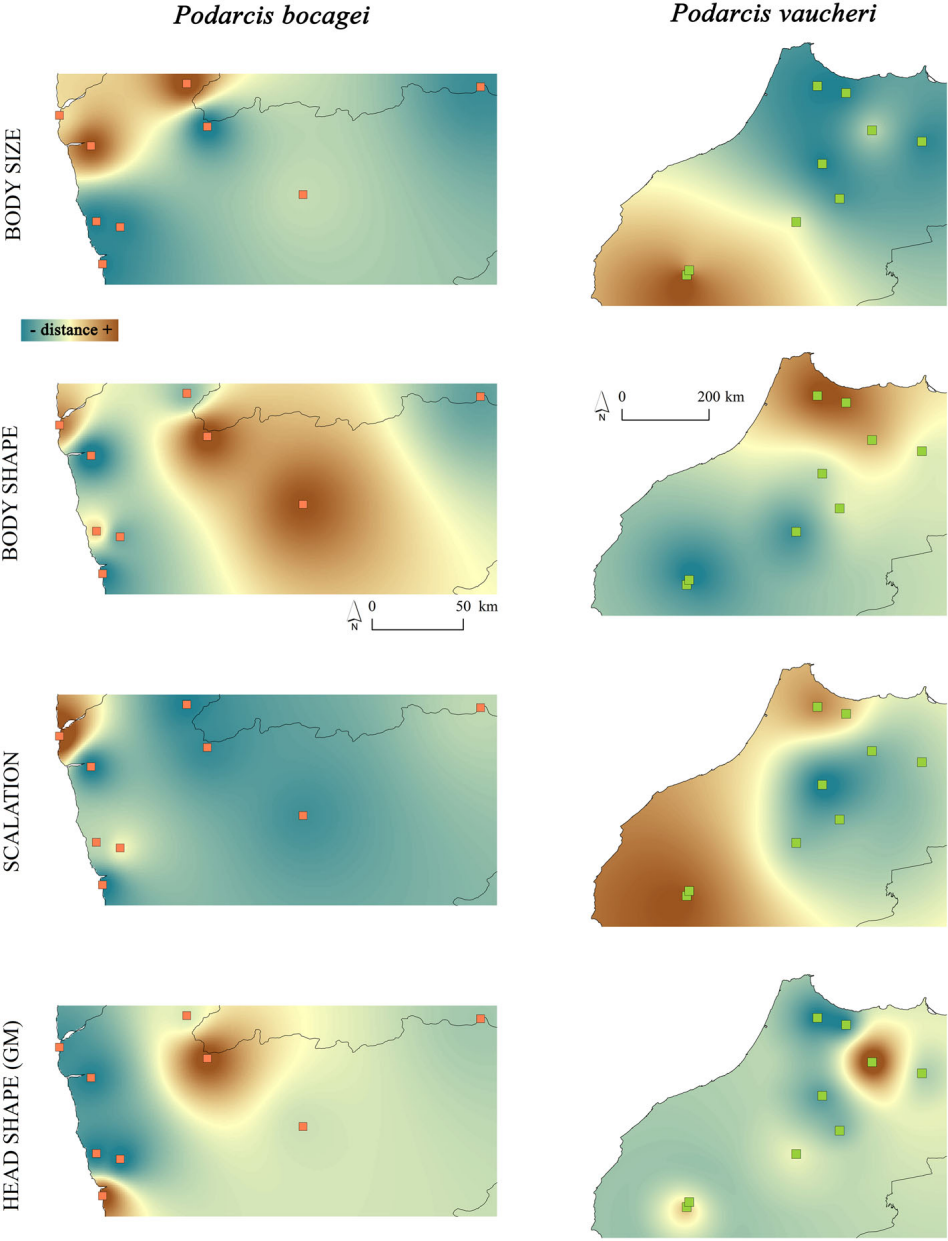
Interpolated phenotypic distances among populations for the two species. For each trait, only accurate rasters displaying spatial variation were considered (see SM 7)

By contrast, phenotypic differentiation among populations exhibited a coherent geographic structure for most studied traits. We globally obtained a reasonable number of accurate rasters to illustrate general patterns of geographic variability, except for the case of scalation traits in *P. bocagei* (three rasters only; Additional file 1: Table A8.2). In both species, some populations were identified as more dissimilar compared to the rest (i.e. they exhibited higher distances in interpolations; Fig. 2), but population divergence patterns varied extensively across traits. For instance, some coastal and inland mountain populations of *P. bocagei* were more dissimilar than the rest in body size, body shape and head shape; while for *P. vaucheri*, Rif and Western High Atlas populations differed from the rest in scalation, body size (High Atlas populations only) and shape (Rif populations only) (Fig. 2).

### Factors shaping morphological variation

**Fig. 3:**
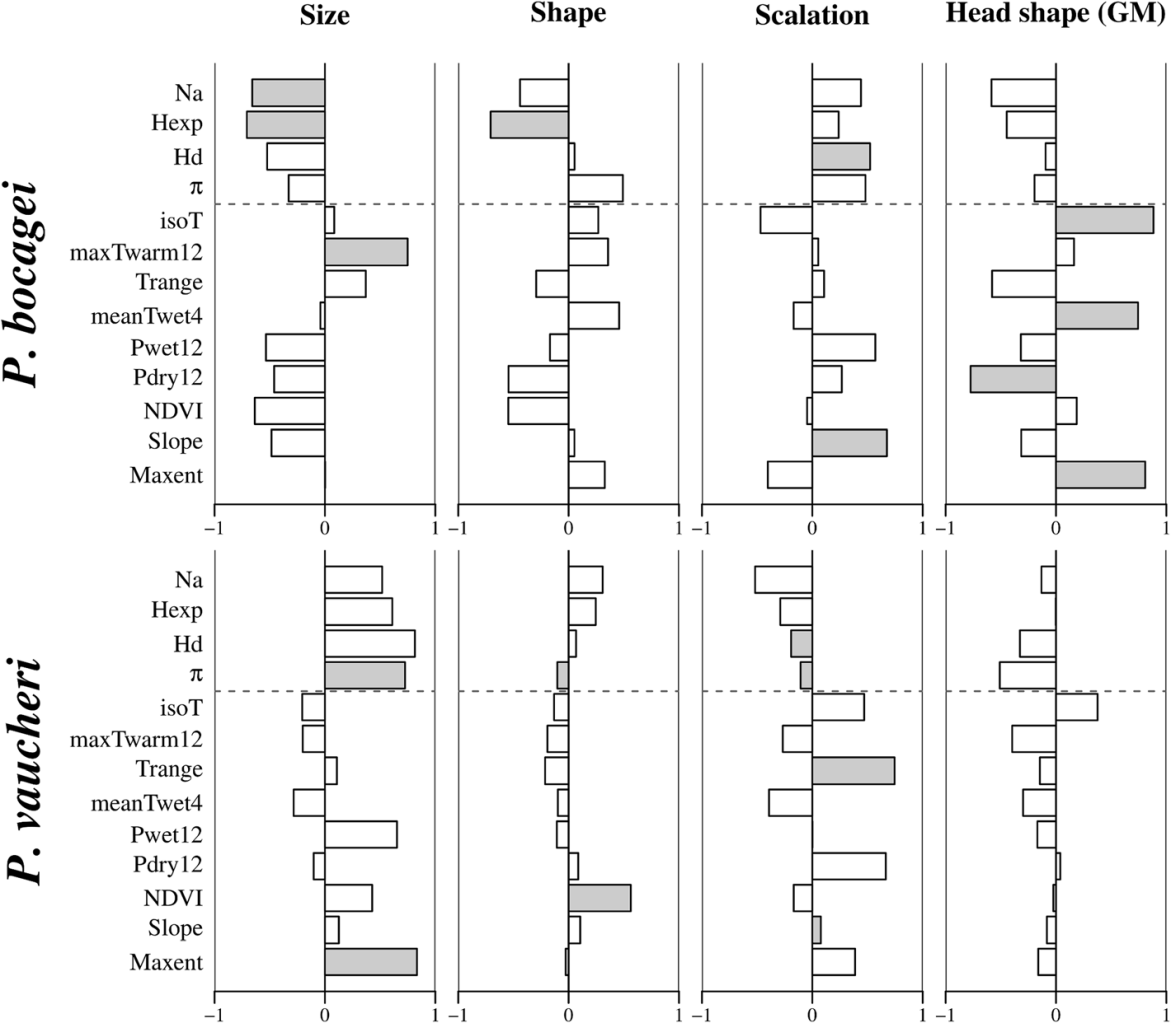
Correlations between multivariate within-population phenotypic diversity and genetic (above the dashed line) and environmental (below the dashed line) explanatory factors. Significant correlations are marked with grey background. See methods for variable abbreviations

Generalized least-squares (GLS) analyses indicated a significant association between levels of within-population phenotypic and genetic diversity, as well as a significant potential contribution of environmental factors (Additional file 2). Levels of population genetic diversity were relevant for explaining phenotypic diversity for all phenotypic traits – except for head shape – in both species. Interestingly, the association between phenotypic and genetic diversity within populations was not always positive. Indeed, while a positive correlation was observed for scalation in *P. bocagei* and body size in *P. vaucheri*, the remaining significant associations (i.e. for body size and shape in *P. bocagei*; and for body shape and scalation in *P. vaucheri*) were negative (Fig. 3). Population phenotypic diversity was also influenced by environmental factors, though such associations varied between species (Fig. 3). Variables related to temperature always had a positive effect on phenotypic diversity, while the effect of precipitation was limited to a negative influence on head shape diversity in *P. bocagei*.

Generalized Dissimilarity Modelling (GDM) indicated that both genetic and environmental factors influence phenotypic differentiation across populations, but the relative contribution of different explanatory variables varied across traits and between species (Fig. 3). Population genetic differentiation at the mtDNA level was associated with the degree of population divergence in scalation in both species, but recent population connectivity, as captured using microsatellite data, did not exhibit a significant contribution. Population divergence in scalation was also influenced by environmental variation across sites and specifically by precipitation in the driest month (Pdry12) and maximum temperature in the warmest month (maxTwarm12), in *P. vaucheri*, but not in *P. bocagei*. Variation across populations in body size and shape was associated to environmental, but not genetic, variation. For body size, slope, mean temperature of the wettest quarter, temperature range and productivity (NDVI) were relevant for explaining population divergence in *P. vaucheri* but not in *P. bocagei*, for which a significant model could not be obtained based on the considered explanatory variables. By contrast, variation across populations of *P. bocagei* in body shape was associated mainly to slope, with an additional significant contribution of geographic distance, whereas precipitation in the driest month was the only variable contributing to explaining variation in body shape in *P. vaucheri*. Finally, divergence across populations in head shape could not be associated to any of the examined genetic or environmental factors in neither species.

## Discussion

Understanding how phenotypic variation is structured across hierarchical levels of biological organization, and determining how it is associated to underlying genetic and environmental factors, is essential for our comprehension of the evolutionary mechanisms that drive biodiversity patterns. Both at the intra- and at the inter-specific levels, neutral evolution through drift-related processes has been predicted to bind genetic and phenotypic variation together, which may translate into common patterns of within-population diversity, among-population divergence and, ultimately, cross-species diversification [24]. At the other extreme of this spectrum, selective mechanisms may cause plastic and adaptive responses that are identifiable as correlations between the environmental niche and phenotypic traits.

### The structure of variation from individuals to populations

The accumulation of phenotypic diversity across hierarchical levels may be predicted to cause concordant patterns of variation across individuals, populations and even species, if the mechanisms driving diversification are uniform across these levels. Our results partially support this idea, as general patterns of morphological variation were found to be uniform across individuals and among populations (Fig. 1). For biometric traits, body size appears as a strong integrating factor, which dominates variation both among individuals and across populations. This is a common and well-known pattern in intraspecific morphological studies, as ontogenetic growth [25], but also possible sampling effects [26], easily yield high levels of size variation, particularly across individuals [27]. Interestingly, the same was the case in our system for scalation, which also exhibited similar structures of variation at the individual and population level, but not for head shape, where principal vectors of variation differed significantly for individual and population-level analyses (Fig. 1). These results add to our understanding of how phenotypic diversity scales up from individuals, through populations, to among species. Previous studies have compared the structure of between intra- and interspecific differentiation, suggesting that concordance at these levels is prone to occur as a response to selection [28]. Our results suggest that this scaling up of morphological variation can also be traced to the individual level, and individual phenotypic variation then translates into divergence among populations, which has been suggested to have potential effects on ecological success [29].

The uniformity in the general direction of morphological variation across hierarchical levels contrasts with its geographic structure, where visibly dissimilar patterns were found when examining divergence across populations and diversity within them. Indeed, while coherent geographic patterns where observed for divergence in most morphological traits (Fig. 2), intra-population diversity levels only exhibited geographic structuring in one trait (i.e. body size; Additional file 1: Table A8.2, Figure A8). This result may be partially related to the covariance structure among individual measured traits, which is generally stronger at the population than at the individual level, as expressed by high eigenvalues concentrated across the first PC axes of population-level analyses. This means that while variation across individuals is more prone to vary in direction across traits, population differentiation in some traits is also accompanied by differentiation in others. Therefore, while principal components of variation are concordant at the individual and population levels, there is no agreement between them in geographic structure. This result suggests that, whatever the mechanisms that underlie the links observed across hierarchical levels of phenotypic diversity, these are not associated to geography. The presence of geographic structure in population divergence, particularly in scalation traits and body shape, is typically evoked as providing evidence for local adaptation or phenotypic plasticity as a response to environmental conditions (e.g. [30, 31], see also below), but the geographic structure of within-population diversity has not been frequently explored – to our knowledge. Based on population dynamics, a dissociation between the amount of within-population diversity and among-population divergence may be expected as a result of reduced effective population sizes: if intra-population diversity is low, divergence among populations may increase due to drift and random phenotypic oscillation, as typically observed in e.g. island populations [32, 33], or during the establishment of invasive populations [34]. Nevertheless, if drift alone drives phenotypic variation, divergence among populations is not expected to exhibit a geographic structure, as that observed in our data. Associations of within-population diversity and among-population divergence with genetic and environmental factors shed more light into the possible causes underlying phenotypic diversification.

### Diversity within populations: Recent phenotypic dynamics

Our results suggest that variation across individuals within populations results from an amalgamation of recent population dynamics and environmental factors. The detailed mechanisms that drive phenotypic variation at the individual level seem to act at a very local scale, as within-population diversity does not exhibit a coherent geographic pattern in most studied traits (Additional file 1: Table A8.2). Also, such mechanisms appear quite labile, as patterns vary extensively across traits and between species (Fig. 3). Population genetic diversity estimated based on microsatellite analyses had a significant effect on phenotypic diversity for all examined traits except for head shape. Such an association between genetic and morphological diversity is expected under several evolutionary scenarios. First, higher genetic diversity may be intuitively expected to be linked to higher phenotypic diversity, as the first would provide the genetic background for the expression of different phenotypes. On the contrary, a negative association can also be observed if a depletion of genetic diversity interferes with basic developmental mechanisms involved in phenotypic regulation [35].

Our results support the idea that developmental mechanisms may be important in shaping levels of phenotypic diversity. In addition to a negative association between genetic and phenotypic diversity, we also found a positive contribution of temperature-related variables. Such an effect may also be mediated by development: as oviparous ectotherms, wall lizards are quite exposed to environmental effects, particularly after oviposition and throughout the post-natal period. Studies of variance within and across individuals in lizards exposed to different environmental conditions have shown that some morphological traits are particularly prone to temperature and other environmental effects during development [36–40], with potential relevance for shaping intrapopulation diversity, as well as population divergence and patterns of geographic variation [41].

### Divergence across populations: Different traits, different stories

Patterns of divergence across populations varied extensively across traits, and were associated to different genetic and environmental factors. For scalation, we found that phylogeographic structure and evolutionary history partially explained divergence among populations in both species. This reinforces the long-established perception that these traits are useful for investigating past and emergent differentiation [23] and for making systematic inferences (e.g. [42]). Still, variation in scalation traits does not seem to be completely neutral, as it has been related to environmental and ecogeographic variation [17, 19]. Indeed, the number of scales has been frequently associated to thermoregulation and water balance in lizards [43, 44], and it is known to co-vary with the thermal and hydric characteristics of the environment, both within [31, 32] and across species [28, 30] in several lizard groups. Accordingly, we found that divergence among populations of *P. vaucheri* in scalation is associated to both temperature and precipitation (Fig. 3), and specifically to conditions that may be limiting for these species, as the variables that significantly contributed to explaining morphological variation represent extremes of environmental factors.

This contribution of restrictive environmental variables, together with the lack of significant effects of environmental factors on scalation divergence in *P. bocagei*, suggest that the magnitude of variation examined here might also be contributing to the observed results. For instance, the distribution range of *P. vaucheri*, and especially the populations examined here, encompass a wider range of environmental conditions [45], which may facilitate the detection of statistically significant effects. This does not seem to be limited to scalation, as significant environmental effects on body size and shape divergence were also identified mostly for populations of *P. vaucheri* (Table 1). However, coherent patterns of geographic variation were identified for all these traits in both species, where some coastal and high-mountain populations exhibited a higher distinctiveness, depending on the trait under examination (Fig. 2). This is in accordance with previous observations in other species of the *P. hispanica* complex (i.e. *P. carbonelli* [18]), and it suggests that possibly other environmental factors, not examined here, or the spatial structuring of genetic variation, may contribute in shaping geographic morphological variation.

**Table 1:** Final models obtained through backward stepwise selection based on Monte Carlo permutations using Generalized Dissimilarity Modelling to explore genetic and environmental factors contributing to phenotypic differentiation among populations of each species

		*P. bocagei*				*P. vaucheri*			
		size	shape	GM	scalation	size	shape	GM	scalation
	% expl	43.14	58.01	0.00	49.42	61.25	43.91	0.00	49.06
	p	0.056	0.001	1.000	0.010	0.001	0.021	1.000	0.013
Microsatellites	Fst								
	genD								
mtDNA	Fst								0.230
	genD				1.000				
Environment	isoT								
	maxTwarm12								0.247
	Trange					0.175			
	meanTwet4					0.199			
	Pwet12								
	Pdry12						1.000		0.523
	NDVI					0.112			
	Slope		0.704			0.514			
	Maxent								
Space	Geographic D		0.296						

% expl: percentage of variance explained by the final model; p: corresponding *p*-value. Numbers below significantly contributing predictor variables describe their relative importance for explaining variance in the response differentiation matrix

On the other hand, head shape divergence does not exhibit an easily explicable geographic pattern, and it could not be associated to either genetic or environmental factors in any of the two species. Local variation observed in our data, as well as geographic gradients not related to environmental differences between sampling sites, suggest that other factors, not investigated here and working at the population level might be important. First, the ecophysical characteristics of the sites sampled may have a contribution. Indeed, head shape is known to exhibit fast evolutionary responses to structural habitat in these lizards, and it varies both across species with different ecological habits [46, 47] and between populations that occupy different microhabitats [48]. Second, developmental processes such as allometry and developmental stability are known to promote fast evolutionary change in head shape throughout ontogeny (e.g. [31, 37]) with important influences on phenotypic diversity [49].

## Conclusions

Overall, our results from two species of wall lizards suggest that establishing simple, global rules to link the phenotype and either genetic or environmental variation is not straightforward. Despite concordant general patterns across hierarchical levels (i.e. individuals and populations), within-population diversity and among-population divergence vary extensively across phenotypic traits, and they exhibit varying levels of geographic structuring and of association to genetic and environmental factors. While some of the traced links comply with ecological predictions and evolutionary intuition, others are difficult to explain in the geographic framework used, suggesting that habitat characteristics and other regulatory mechanisms – not examined here –, may be more influential in shaping patterns of phenotypic diversity.

## Methods

### Model system

*Podarcis* wall lizards are an intriguing model for exploring how variation scales from individuals to populations, eventually determining diversity across species. Ranging around the Mediterranean Basin, species of this genus occupy a wide variety of ecosystems [50]. Their morphology has been shown to respond to different characteristics of the environment including habitat structure [46–48], insularity [51, 52], and human-mediated disturbance [36, 37]. Further, geographically-structured morphological variation has been reported within some species (e.g. [18]), but the causes underlying such structuring have not been explored.

**Fig. 4:**
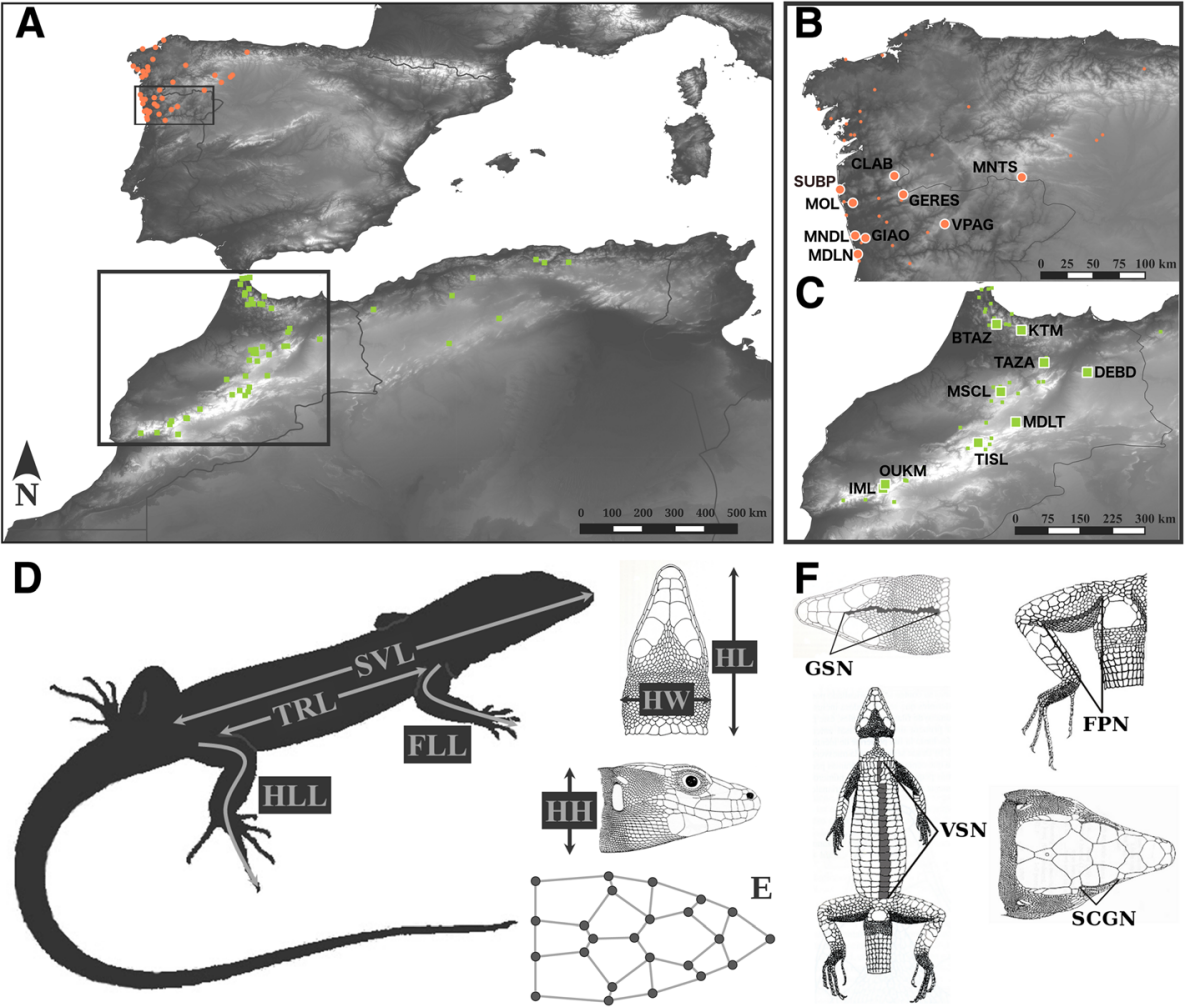
Global known distribution of *Podarcis bocagei* (red dots) and *P. vaucheri* from Morocco and Algeria (green squares) (**a**; modified from Kaliontzopoulou et al. 2011) and localization of the sampled populations (**b**: *P. bocagei*, **c**: *P. vaucheri*; in larger symbols, with white outline, codes as in SM 1), and measured biometric traits (**d**), landmarks used for head shape analyses (**e**) and counted scalation characters (**f**). See methods for variable abbreviations. The black squares in the global map (**a**) denote the areas used for interpolating morphological traits in each of the species

Here, we investigated two species of wall lizards, *Podarcis bocagei* from Northwestern Iberia, and *P. vaucheri* from Morocco and Algeria (sensu [22]). For each of these species, we sampled a total of nine populations (Fig. 4, Additional file 3), in the years between 2005 and 2009. In each population, we captured around 30 adult individuals of both sexes (Additional file 3). For each individual, we recorded several biometric traits (see below); we took high-resolution photographs to record head shape using geometric morphometrics, and to quantify scalation traits; and we removed a small tip of the tail, which was stored in 96% ethanol to be used for genetic analyses. All individuals were processed in the field and released immediately after.

### Morphological variation

To characterize morphological diversity within and divergence across populations, we considered four sets of traits: body size, body shape, head shape using geometric morphometrics, and scalation. We chose these traits due to their relevance for our hypotheses, as they may be associated to both stochastic (genetic) and adaptive (environmental) factors. Body size, body shape and head shape are known to be of relevance for a series of physiological, ecological and social functions in lizards. On the other hand, scale counts are frequently related to environmental variability, showing geographic variation [17, 19], but they have also been extensively used for systematics (e.g. [42]), which suggests at least some association to genetic divergence.

To quantify body size and shape, we recorded seven linear biometric traits to the closest 0.01 mm using electronic calipers: snout-vent length, trunk length, head dimensions (i.e. length, width and height), and fore and hind limb length (Fig. 4). These traits capture the general biometric properties of each individual and they are associated to whole-organism performance [53, 54], thus being potentially associated to environmental variation. Because *Podarcis* wall lizards exhibit marked sexual dimorphism in body size and shape [55, 56], we first centered all observations to the corresponding sex-mean. The first principal component of the matrix of sex-mean-centered biometric traits was then treated as a measure of body size, while the remaining PC axes were treated as a multivariate, size-corrected measure of body shape (see also results).

Because head shape has been shown to be an important component of ecologically-related variation in these lizards (i.e. [46, 48]), we also used geometric morphometrics (GM) to quantify variation in the position of 24 two-dimensional landmarks on the dorsal head surface (Fig. 4). A detailed account of all GM data processing can be found in Additional file 4.

To capture variation in scalation, in each individual we counted the number of gular (GSN) and ventral (VSN) scales, femoral pores (FPN), and supraciliary granules (SCGN) (Fig. 4). These traits were chosen because they are all continuously valued, and they exhibit enough variation across individuals of *Podarcis* to be informative (i.e. they are not fixed [23]). All scalation traits were sex-mean-centered prior to statistical analyses.

Based on each of the above sets of traits, we quantified the level of diversity within each population using the mean distance to the population centroid [57]. In addition, we represented the degree of morphological divergence among populations of each species by calculating multivariate (or univariate, in the case of body size), pairwise Euclidean distances between population mean values. All raw data used to calculate diversity within and divergence between populations are available in Additional file 5 (*P. bocagei*) and Additional file 6 (*P. vaucheri*).

### Population genetics and phylogeographic structure

To characterize recent population dynamics, we genotyped all individuals examined for morphological analyses for nine microsatellite loci, initially developed for *P. bocagei* [58]. Based on the data obtained, we characterized within-population genetic diversity using the number of alleles per population (Na) and expected heterozygosity (Hexp). We also characterized differentiation between populations by calculating the Cavalli-Sforza and Edwards (1967) [59] genetic distance (genD, with a correction for null alleles, see Additional file 7) and Fst values according to [60].

In addition, we examined a 623 bp fragment of the NADH dehydrogenase subunit 4 (ND4) mtDNA gene to describe the phylogeographic structure and deeper evolutionary history of the populations under study. Based on these data, we described the genetic distance among populations using Dxy, the average number of nucleotide substitutions per site between populations [61]; and estimated differentiation using the Hudson et al. (1992) estimator of Fst [62], and calculated diversity measures per locality (haplotype diversity, Hd, and nucleotide diversity, π).

A detailed account of laboratory and analytical procedures followed to obtain genetic data, and the corresponding descriptors of population diversity and differentiation, are provided in Additional file 7. Raw individual multilocus genotypes for microsatellite data are available in Additional file 8.

### Environmental variation

To characterize the average climatic environment of the studied populations, and fluctuations to it, we clipped the 19 bioclimatic variables (at 30 arc-seconds of resolution) included in the Worldclim database [16] to our study area. To avoid variable collinearity, we reduced this set to six variables that exhibited low correlations (*R* < 0.7), and which are known to be biologically informative for the species under study [45, 63], including: isothermality (isoT), maximum temperature of the warmest month (maxTwarm12), annual range of temperature (Trange), mean temperature of the wettest quarter (meanTwet4), precipitation of the wettest and driest month (Pwet12 and Pdry12, correspondingly). In addition to these climatic variables, and in order to characterize the local productivity of habitats occupied by wall lizards, we extracted the values of a Normalized Difference Vegetation Index (NDVI) for each locality (see [64]). We obtained these values by averaging the satellite-derived temporal raster series of the MODIS NDVI imagery (from GIMMS Global Agricultural Monitoring System, http://glam1.gsfc.nasa.gov/; at 250 m of resolution) for the presumed activity period of wall lizards during our sampling periods (late February to early November, in the years between 2005 and 2009). Furthermore, to characterize topographic conditions of each locality we derived the slope variable from altitude (also downloaded from the Worldclim database), using the “slope” function of ArcGIS [65]. Finally, we quantified environmental suitability for each of the studied populations extracting values from ecological niche-based models (ENM) calculated for the whole range of each species using the Maxent software [66]. Further details on ENMs can be consulted in Additional file 9.

### The structure of morphological variation

To investigate whether the structure of morphological variation was concordant across individuals and across populations of each species, we performed principal components analyses of each multivariate trait block separately (i.e. biometry, head shape, and scalation), considering a) the matrix of individual observations and b) population means for each trait. We then permuted population-centered residuals of individual observations across populations to examine whether the first principal component of individual morphological variation aligned with that of population divergence, by testing whether the angle θ between these two PC vectors was larger than that expected at random.

### Modelling diversity within populations

To visualize the spatial structure of morphological diversity within populations, we spatially interpolated within-population mean distance to centroid for each of the four explored trait sets for each species, using the kriging interpolation method in ArcGIS [46]. A detailed description of the methods used for interpolations is available in Additional file 1.

To evaluate which genetic and environmental factors may contribute in shaping levels of intrapopulation phenotypic diversity, we used Generalized Least Squares (GLS) to fit single-predictor linear models while taking the spatial structure of our sampling into account. Specifically, we estimated GLS regressions with population phenotypic diversity (i.e. mean distance to the centroid, see above) for each of the morphological traits examined as the response; and each of the genetic diversity and environmental factors described above as predictors. We preferred this simple, pairwise approach, instead of fitting multiple-predictor GLS models, to obtain an overall view of which factors may influence intraspecific phenotypic diversity without confounding statistical interactions among predictors.

The most adequate autocorrelation structure for each response-predictor combination was determined by fitting models using a linear, exponential, Gaussian, or rational quadratic structure, and then comparing their fit to the data using Akaike’s Information Criterion (AIC). The model with the lowest AIC for each combination was considered to optimize the expected covariance among observations due to spatial proximity. When GLS models suggested a significant contribution of a genetic or environmental predictor on the level of population diversity for a trait, we examined the strength of such an association by inspecting the Pearson correlation between response and predictor variables.

### Modelling divergence across populations

Similar to what we did for intra-population diversity, we used kriging of inter-population phenotypic distances to explore the geographic structure of phenotypic divergence among populations (Additional file 1). In continuation, we used Generalized Dissimilarity Modelling (GDM) [67] to investigate whether recent population connectivity, evolutionary history, and/or environmental factors contribute in explaining morphological divergence across populations, while taking spatial structure into account. This technique allows to model dissimilarities in morphology, represented as the matrix of pairwise distances between population means, with pairwise matrices representing geographic proximity, genetic differentiation, and environmental dissimilarity across populations. It is particularly suitable for our purposes, because (1) it facilitates the application of regression-type analyses on data that are naturally expressed as distances (e.g. genetic differentiation); (2) it allows the inclusion of non-linear relationships between response and explanatory dissimilarity matrices [68]; and (3) by working with multivariate distance matrices, one can evaluate the sources of divergence in multivariate phenotypes examining the response of several phenotypic traits simultaneously, similarly to approaches implemented to genomic data [69].

We fit GDMs to inter-population Euclidean distance matrices for each of the investigated phenotypic traits and for each species separately using the gdm package for R [70]. Predictors included the matrices of Fsts and genetic distances, based on microsatellite (recent population connectivity) and mtDNA data (evolutionary history); the nine environmental variables described above; and the matrix of pairwise geographic distances between sampling sites. We performed a backward-stepwise selection of variables using Monte Carlo permutations to evaluate predictor significance [67, 71]. To quantify the relative contribution of each predictor in explaining variance in phenotypic differentiation for each of the response traits we summed the coefficients of the I-splines, which corresponds to the maximum height attained by the response curve for each predictor [67, 69].

To account for the possibility that environmental resistance, instead of geographic distance, may best represent the expected covariance among populations for phenotypic traits, we repeated all GDM analyses considering pairwise matrices of environmental connectivity, calculated in Circuitscape [72] based on the inverse of habitat suitability calculated for each species using ENMs in Maxent (see above, and Additional file 9). However, because the results obtained did not vary substantially when using connectivity instead of geographic distance, we only present the results using geographic distances.

## Additional files

Additional file 1: Interpolation of morphological diversity within and differentiation across populations, provides details on the methods used and the results obtained to study the geographic structure of morphological variation. (DOCX 169 kb)

Additional file 2: Results of Generalized Least Squares analysis performed to identify environmental and genetic variables that contribute significantly in explaining differences across populations in levels of intra-populational morphological diversity while taking geographic distance into account. (DOCX 73 kb)

Additional file 3: Geographic coordinates in the WGS1984 system, number of individuals sampled per sex (Nm: males, Nf: females) and in total (Ntot), and abbreviation (CODE) for the examined populations. (DOCX 16 kb)

Additional file 4: Geometric morphometric procedures for quantifying variation in dorsal head shape, provides a detailed description of all steps involved in obtaining head shape data, including landmark definitions and digitizing, superimposition of landmark coordinates and other pre-processing applied to GM data. (DOCX 13 kb)

Additional file 5: Sex-corrected individual values for all morphological traits for populations of *P. bocagei*, provides the data used for all analyses for this species, after mean-centering by sex, including biometric and scalation variables as described in Methods, and superimposed, sex-mean-centered landmark coordinates (X1 – Y24), as well as the population of origin in accordance with Additional file 3. (TXT 218 kb)

Additional file 6: Sex-corrected individual values for all morphological traits for populations of *P. vaucheri*, provides the data used for all analyses for this species, after mean-centering by sex, including biometric and scalation variables as described in Methods, and superimposed, sex-mean-centered landmark coordinates (X1 – Y24), as well as the population of origin in accordance with Additional file 3. (TXT 203 kb)

Additional file 7: Genetic analyses, provides a detailed account of of laboratory and analytical procedures followed to obtain genetic data, and the corresponding descriptors of population diversity and differentiation. (DOCX 81 kb)

Additional file 8: Individual multilocus genotypes for the nine microsatellite loci. Each allele is coded as its length, given in bp. Missing data is represented by “0”. (XLSX 66 kb)

Additional file 9: Maxent models for quantifying environmental suitability, provides a description of methods and results related to ENM used to obtain habitat suitability maps for each species. (DOCX 727 kb)

## Abbreviations

AIC: Akaike’s Information Criterion
Dxy: Average number of nucleotide substitutions per site between populations
ENMs: Ecological niche-based models
FLL: Fore-limb length
FPN: Number of femoral pores
GDM: Generalized Dissimilarity Modelling
genD: Genetic distance
GLS: Generalized Least-Squares
GM: Geometric morphometrics
GSN: Number of gular scales
Hd: Haplotype diversity
Hexp: Expected heterozygosity
HH: Head height
HL: Head length
HLL: Hind-limb length
HW: Head width
isoT: isothermality
maxTwarm12: Maximum temperature in the warmest month
meanTwet4: Mean temperature of the wettest quarter
Na: Number of alleles
NDVI: Normalized Difference Vegetation Index
PC: Principal components
Pdry12: Precipitation in the driest month
Pwet12: precipitation of the wettest month
SCGN: Number of supraciliary granules
SVL: Snout-vent length
Trange: Annual range of temperature
TRL: Trunk length
VSN: Number of ventral scales
π: Nucleotide diversity
