# Supplementary material for: Where does diversity come from? Linking geographical patterns of morphological, genetic, and environmental variation in wall lizards
Source: BMC Evol Biol. 2018 Aug 22;18:124. doi: 10.1186/s12862-018-1237-7 (PMC6113677; doi:10.1186/s12862-018-1237-7)
Supplement: Supplementary file 1 — Interpolation of morphological diversity within and differentiation across populations, provides details on the methods used and the results obtained to study the geographic structure of morphological variation. (DOCX 169 kb) [file 12862_2018_1237_MOESM1_ESM.docx]

**Additional file 8:** Interpolation of morphological diversity within and differentiation across populations

In order to explore spatial structure in diversity within populations, we interpolated within-population diversity values for each of the four studied sets of traits (body size and shape, head shape and scalation), as quantified using the mean distance to the centroid (see Methods). Similarly, to visualize spatial patterns of morphological divergence across populations, we spatially interpolated multivariate distance values among populations of each species for the same four sets of traits.

Interpolation allows predicting values for cells in a raster from a limited number of sample data points, assuming that values of data points are continuous and spatially autocorrelated (ESRI, 2014). We used the kriging interpolation method based on a spherical model (Oliver 1990), implemented in the ‘Geostatistical Analyst’ extension of ArcMap (Johnston et al. 2001). Accuracy of interpolations was measured by the mean error, which should be close to 0, by the root mean-square error, which should be the smallest possible, and by the average standard error and the root-mean-square standardized error, which should be close to 1 (for details see Johnston et al. 2001).

For diversity within populations, a georeferenced database containing values for each trait was imported into ArcGIS. Values for each trait were then interpolated to obtain spatial surfaces of variation in diversity. For differentiation across populations, georeferenced distance matrices for each trait were imported to ArcGIS, where distances of one population to the remaining eight populations of the same species were interpolated. This process was performed nine times by trait and species, as each species included nine populations for each of the four traits. In some cases interpolation was rejected due to low spatial autocorrelation of the data (detected when the semivariogram was visualized; Oliver 1990). As such, interpolated rasters with low accuracy or lack of spatial variation were discarded. The remaining rasters were summed to visualize the general pattern of geographical variation for each trait and species. Interpolated surfaces were visualized to verify their variation and identify the occurrence, or not, of spatial patterns. In both differentiation across and diversity within populations, error statistics were used to evaluate the accuracy of kriging interpolations (Tables A7.1 and A7.2).

**References not included in the main text**

Johnston K, Ver Hoef JM, Krivoruchko K, Lucas N (2001) Using ArcGIS Geostatistical Analyst. Environmental Systems Research Institute, Inc., Redlands.

Oliver MA (1990) Kriging: a method of interpolation for geographical information systems. Int J Geogr Inf Syst 4:313–332.

**Table A8.1:** Error metrics for kriging interpolations of the four examined within-population diversity traits for populations of the two species. Occurrence or not of spatial pattern is depicted.

| ***P. bocagei*** | | | | |
| --- | --- | --- | --- | --- |
| **Error metric** | **Body size** | **Body shape** | **Scalation** | **Head shape** |
| Mean | -0.006 | -0.001 | 0.000 | 0.000 |
| Root-mean-square | 0.026 | 0.018 | 0.015 | 0.000 |
| Mean standardized | -0.123 | -0.032 | -0.015 | 0.028 |
| Root-mean-square standardized | 1.016 | 1.055 | 0.996 | 1.007 |
| Avg standard error | 0.024 | 0.017 | 0.015 | 0.000 |
| Spatial pattern | YES | NO | NO | NO |
| ***P. vaucheri*** | | | | |
| **Error metric** | **Body size** | **Body shape** | **Scalation** | **Head shape** |
| Mean | 0.000 | 0.000 | 0.000 | 0.000 |
| Root-mean-square | 0.030 | 0.022 | 0.021 | 0.000 |
| Mean standardized | 0.001 | 0.000 | 0.000 | 0.000 |
| Root-mean-square standardized | 1.004 | 1.000 | 1.072 | 1.000 |
| Avg standard error | 0.029 | 0.022 | 0.020 | 0.000 |
| Spatial pattern | YES | NO | NO | NO |

**
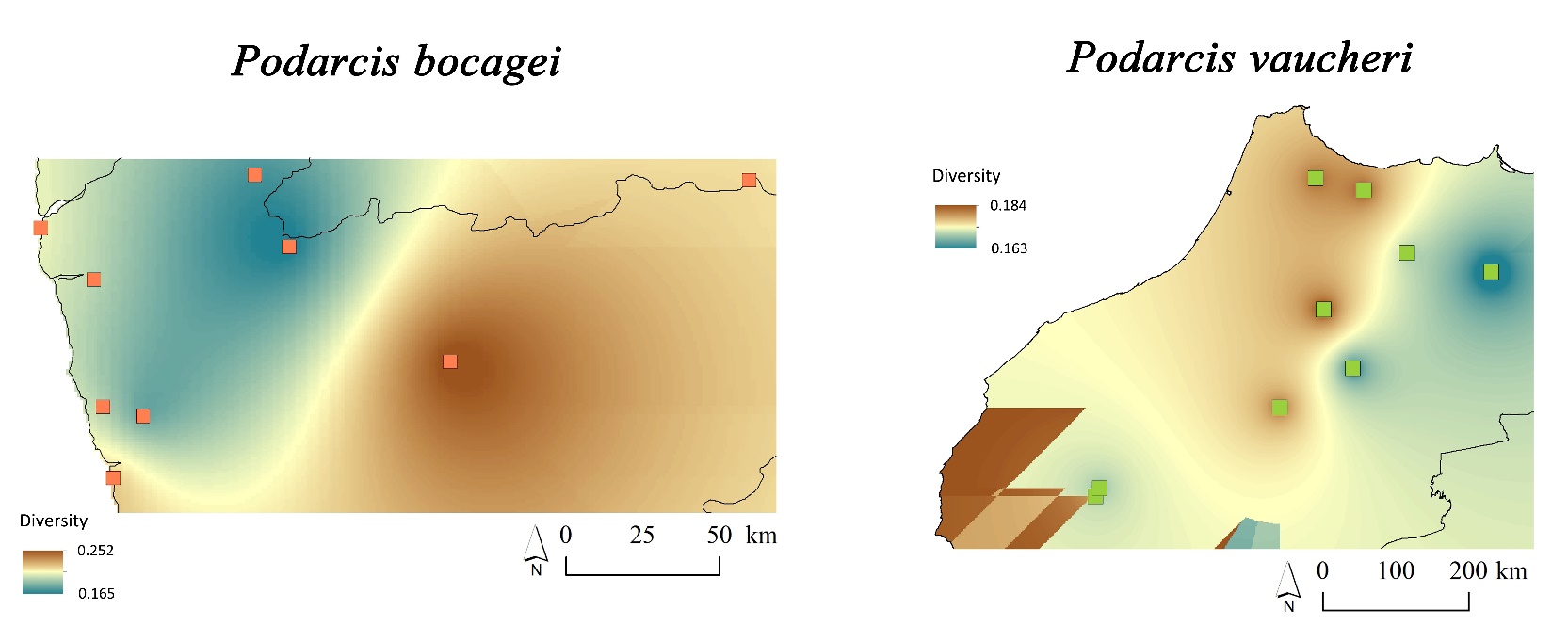
**

**Figure A8.** Geographic variation of diversity in body size within populations of *Podarcis bocagei* and *P. vaucheri.*

| **Table A8.2:** Average (and standard deviation) error metrics for kriging interpolations of the four examined multivariate trait distances among populations of the two species, and number of accurate rasters showing spatial variation that were used to visualize spatial patterns. | | | | |
| --- | --- | --- | --- | --- |
| ***P. bocagei*** | | | | |
| **Error metric** | **Body size** | **Body shape** | **Scalation** | **Head shape** |
| Mean | 0.005 (0.014) | 0.0001 (0.002) | 0.001 (0.001) | 0.0001 (0.001) |
| Root-mean-square | 0.037 (0.007) | 0.03 (0.006) | 0.042 (0.006) | 0.006 (0.001) |
| Mean standardized | 0.023 (0.023) | 0.012 (0.041) | 0.005 (0.017) | 0.021 (0.048) |
| Root-mean-square standardized | 0.912 (0.099) | 1.072 (0.172) | 1.012 (0.037) | 1.065 (0.233) |
| Avg standard error | 0.041 (0.007) | 0.028 (0.004) | 0.042 (0.007) | 0.006 (0.001) |
| N rasters | 6 | 6 | 3 | 4 |
| ***P. vaucheri*** | | | | |
| **Error metric** | **Body size** | **Body shape** | **Scalation** | **Head shape** |
| Mean | 0.001 (0.002) | 0.000 (0.001) | 0.000 (0.001) | 0.000 (0.0002) |
| Root-mean-square | 0.072 (0.018) | 0.047 (0.01) | 0.054 (0.007) | 0.01 (0.001) |
| Mean standardized | 0.009 (0.02) | 0.001 (0.015) | 0.002 (0.009) | 0.007 (0.01) |
| Root-mean-square standardized | 1.046 (0.064) | 0.986 (0.03) | 1.077 (0.051) | 1.002 (0.043) |
| Avg standard error | 0.069 (0.017) | 0.047 (0.011) | 0.04 (0.007) | 0.01 (0.001) |
| N rasters | 6 | 7 | 7 | 5 |
