# Supplementary material for: Where does diversity come from? Linking geographical patterns of morphological, genetic, and environmental variation in wall lizards
Source: BMC Evol Biol. 2018 Aug 22;18:124. doi: 10.1186/s12862-018-1237-7 (PMC6113677; doi:10.1186/s12862-018-1237-7)
Supplement: Supplementary file 2 — Results of Generalized Least Squares analysis performed to identify environmental and genetic variables that contribute significantly in explaining differences across populations in levels of intra-populational morphological diversity while taking geographic distance into account. (DOCX 73 kb) [file 12862_2018_1237_MOESM2_ESM.docx]

**Additional file 9:** Results of Generalized Least Squares analysis performed to identify environmental and genetic variables that contribute significantly in explaining differences across populations in levels of intra-populational morphological diversity while taking geographic distance into account.

| **Table A9.1:** Regression coefficients (Value) and their standard error (SE) for each combination of response and predictor variables in *Podarcis bocagei*, and corresponding t- and p-values under GLS with the correlation structure (corStruct) inferred through model comparison (see Table A6.3). Variables with a significant effect are marked in bold letter. Please see Material and Methods for abbreviations of predictor variables. | | | | | | |
| --- | --- | --- | --- | --- | --- | --- |
| **Response** | **Predictor** | **Value** | **SE** | **t-value** | **p-value** | **corStruct** |
| Size | (Intercept) | 0.354 | 0.049 | 7.188 | 1.79*10^-04^ | Gaussian |
|  | **Na** | **-0.010** | **0.003** | **-3.011** | **0.020** |  |
|  | (Intercept) | 1.828 | 0.402 | 4.549 | 0.003 | Exponential |
|  | **Hexp** | **-1.851** | **0.461** | **-4.019** | **0.005** |  |
|  | (Intercept) | 0.223 | 0.015 | 16.134 | 8.55*10^-07^ | Gaussian |
|  | Hd | -0.011 | 0.005 | -1.963 | 0.090 |  |
|  | (Intercept) | 0.235 | 0.016 | 13.787 | 2.49*10^-06^ | Gaussian |
|  | π | -0.014 | 0.006 | -1.673 | 0.138 |  |
|  | (Intercept) | 0.222 | 0.014 | 0.237 | 0.819 | Gaussian |
|  | isoT | -0.041 | 0.021 | 0.749 | 0.478 |  |
|  | (Intercept) | 0.222 | 0.016 | -1.297 | 0.236 | Linear |
|  | **maxTwarm12** | **-11.622** | **6.947** | **2.983** | **0.020** |  |
|  | (Intercept) | 0.051 | 0.213 | 2.234 | 0.061 | Exponential |
|  | Trange | 0.393 | 0.524 | 0.953 | 0.372 |  |
|  | (Intercept) | -0.155 | 0.119 | 6.583 | 3.09*10^-04^ | Gaussian |
|  | meanTwet4 | 0.015 | 0.005 | -0.080 | 0.938 |  |
|  | (Intercept) | 0.143 | 0.064 | 5.005 | 0.002 | Linear |
|  | Pwet12 | 0.003 | 0.003 | -1.721 | 0.129 |  |
|  | (Intercept) | 0.208 | 0.032 | 7.287 | 1.65*10^-04^ | Linear |
|  | Pdry12 | -3.277*10^-04^ | 0.004 | -1.627 | 0.148 |  |
|  | (Intercept) | 0.219 | 0.014 | 8.778 | 5.02*10^-05^ | Exponential |
|  | NDVI | -0.005 | 0.003 | -2.177 | 0.066 |  |
|  | (Intercept) | 0.305 | 0.061 | 15.933 | 9.31*10^-07^ | Gaussian |
|  | slope | -0.001 | 0.000 | -1.614 | 0.151 |  |
|  | (Intercept) | 0.269 | 0.037 | 6.093 | 4.94*10^-04^ | Gaussian |
|  | Maxent | -0.003 | 0.002 | 0.730 | 0.489 |  |
| Shape | (Intercept) | 0.189 | 0.031 | 3.483 | 0.010 | Linear |
|  | Na | 0.042 | 0.057 | -1.196 | 0.271 |  |
|  | (Intercept) | 0.263 | 0.030 | 3.044 | 0.019 | Linear |
|  | **Hexp** | **-0.115** | **0.053** | **-2.643** | **0.033** |  |
|  | (Intercept) | 0.159 | 0.046 | 11.688 | 7.59*10^-06^ | Gaussian |
|  | Hd | -0.004 | 0.003 | 0.262 | 0.801 |  |
|  | (Intercept) | 0.792 | 0.260 | 13.685 | 2.62*10^-06^ | Gaussian |
|  | π | -0.787 | 0.298 | 1.637 | 0.146 |  |
|  | (Intercept) | 0.099 | 0.007 | 0.495 | 0.636 | Linear |
|  | isoT | 0.005 | 0.004 | 0.860 | 0.418 |  |
|  | (Intercept) | 0.100 | 0.013 | 0.164 | 0.874 | Linear |
|  | maxTwarm12 | 0.002 | 0.006 | 1.032 | 0.336 |  |
|  | (Intercept) | 0.103 | 0.009 | 4.122 | 0.004 | Linear |
|  | Trange | 0.005 | 0.018 | -0.844 | 0.426 |  |
|  | (Intercept) | 0.097 | 0.007 | 6.980 | 2.15*10^-04^ | Linear |
|  | meanTwet4 | 7.825 | 4.778 | 1.472 | 0.184 |  |
|  | (Intercept) | 0.038 | 0.077 | 3.089 | 0.018 | Gaussian |
|  | Pwet12 | 0.161 | 0.188 | -0.431 | 0.680 |  |
|  | (Intercept) | 0.014 | 0.087 | 9.060 | 4.09*10^-05^ | Linear |
|  | Pdry12 | 0.004 | 0.004 | -1.856 | 0.106 |  |
|  | (Intercept) | 0.121 | 0.039 | 8.720 | 5.24*10^-05^ | Gaussian |
|  | NDVI | -9.538*10^-05^ | 2.214*10^-04^ | -1.961 | 0.091 |  |
|  | (Intercept) | 0.131 | 0.032 | 11.831 | 6.99*10^-06^ | Gaussian |
|  | slope | -0.001 | 0.002 | 0.397 | 0.704 |  |
|  | (Intercept) | 0.087 | 0.013 | 6.799 | 2.54*10^-04^ | Linear |
|  | Maxent | 0.002 | 0.002 | 1.028 | 0.338 |  |
| GM | (Intercept) | 0.149 | 0.008 | 12.414 | 5.06*10^-06^ | Gaussian |
|  | Na | -4.410*10^-04^ | 0.001 | -1.593 | 0.155 |  |
|  | (Intercept) | 0.113 | 0.018 | 2.386 | 0.048 | Gaussian |
|  | Hexp | 1.846*10^-04^ | 1.003*10^-04^ | -1.120 | 0.300 |  |
|  | (Intercept) | 0.139 | 0.009 | 59.784 | 2.04*10^-10^ | Gaussian |
|  | Hd | 3.346*10^-04^ | 4.565*10^-04^ | -0.294 | 0.777 |  |
|  | (Intercept) | 0.140 | 0.003 | 58.466 | 1.12*10^-10^ | Gaussian |
|  | π | 0.002 | 0.001 | -0.951 | 0.373 |  |
|  | (Intercept) | 0.154 | 0.007 | 6.517 | 3.29*10^-04^ | Linear |
|  | **isoT** | **-0.015** | **0.013** | **4.856** | **0.002** |  |
|  | (Intercept) | 0.147 | 0.012 | 3.275 | 0.014 | Linear |
|  | maxTwarm12 | -0.003 | 0.020 | 1.219 | 0.262 |  |
|  | (Intercept) | 0.035 | 0.003 | 17.905 | 4.19*10^-07^ | Linear |
|  | Trange | -3.067*10^-04^ | 1.925*10^-04^ | -1.831 | 0.110 |  |
|  | (Intercept) | 0.058 | 0.024 | 42.383 | 1.06*10^-09^ | Linear |
|  | **meanTwet4** | **-0.031** | **0.028** | **2.812** | **0.026** |  |
|  | (Intercept) | 0.031 | 0.001 | 11.678 | 7.63*10^-06^ | Rational Quadratic |
|  | Pwet12 | -2.398*10^-04^ | 2.791*10^-04^ | -0.696 | 0.509 |  |
|  | (Intercept) | 0.031 | 0.001 | 41.925 | 1.146*10^-09^ | Linear |
|  | **Pdry12** | **-1.596*10^-04^** | **3.365*10^-04^** | **-3.114** | **0.017** |  |
|  | (Intercept) | 0.018 | 0.003 | 25.520 | 3.62*10^-08^ | Gaussian |
|  | NDVI | 0.032 | 0.007 | 0.621 | 0.554 |  |
|  | (Intercept) | 0.031 | 0.001 | 54.157 | 1.92*10^-10^ | Gaussian |
|  | slope | -3.125*10^-04^ | 0.001 | -0.552 | 0.598 |  |
|  | (Intercept) | 0.031 | 0.001 | 47.217 | 4.99*10^-10^ | Gaussian |
|  | **Maxent** | **-0.314** | **0.330** | **3.535** | **0.010** |  |
| Scalation | (Intercept) | 0.130 | 0.014 | 4.102 | 0.005 | Gaussian |
|  | Na | -0.001 | 0.001 | 1.298 | 0.235 |  |
|  | (Intercept) | 0.102 | 0.009 | 0.064 | 0.951 | Gaussian |
|  | Hexp | 0.001 | 0.002 | 0.650 | 0.536 |  |
|  | (Intercept) | 0.092 | 0.013 | 27.152 | 2.36*10^-08^ | Exponential |
|  | **Hd** | **0.025** | **0.024** | **2.862** | **0.024** |  |
|  | (Intercept) | 0.133 | 0.015 | 34.862 | 4.15*10^-09^ | Exponential |
|  | π | -0.053 | 0.027 | 1.453 | 0.189 |  |
|  | (Intercept) | 0.111 | 0.027 | 5.137 | 0.001 | Gaussian |
|  | isoT | 0.002 | 0.002 | -1.405 | 0.203 |  |
|  | (Intercept) | 0.013 | 0.204 | 2.563 | 0.037 | Gaussian |
|  | maxTwarm12 | 0.152 | 0.234 | 0.143 | 0.890 |  |
|  | (Intercept) | 0.138 | 0.004 | 7.519 | 1.35*10^-04^ | Gaussian |
|  | Trange | 0.006 | 0.002 | 0.283 | 0.785 |  |
|  | (Intercept) | 0.120 | 0.009 | 18.570 | 3.26*10^-07^ | Gaussian |
|  | meanTwet4 | 0.009 | 0.001 | -0.455 | 0.663 |  |
|  | (Intercept) | 0.136 | 0.005 | 6.407 | 3.65*10^-04^ | Gaussian |
|  | Pwet12 | 0.024 | 0.008 | 1.841 | 0.108 |  |
|  | (Intercept) | 0.141 | 0.004 | 15.033 | 1.38*10^-06^ | Gaussian |
|  | Pdry12 | 4.914 | 3.381 | 0.733 | 0.487 |  |
|  | (Intercept) | 0.140 | 0.019 | 12.689 | 4.37*10^-06^ | Gaussian |
|  | NDVI | 2.520*10^-04^ | 0.001 | -0.125 | 0.904 |  |
|  | (Intercept) | 0.200 | 0.039 | 42.732 | 1.00*10^-09^ | Gaussian |
|  | **slope** | **-0.132** | **0.094** | **2.423** | **0.046** |  |
|  | (Intercept) | 0.138 | 0.054 | 20.583 | 1.60*10^-07^ | Gaussian |
|  | Maxent | 3.175*10^-04^ | 0.002 | -1.166 | 0.282 |  |

| **Table A9.2:** Regression coefficients (Value) and their standard error (SE) for each combination of response and predictor variables in *Podarcis vaucheri*, and corresponding t- and p-values under GLS with the correlation structure (corStruct) inferred through model comparison (see Table S6.4). Variables with a significant effect are marked in bold letter. Please see Material and Methods for abbreviations of predictor variables. | | | | | | | | | | |  |
| --- | --- | --- | --- | --- | --- | --- | --- | --- | --- | --- | --- |
| **Response** | **Predictor** | **Value** | **SE** | | **t-value** | | **p-value** | | **corStruct** | |  |
| Size | (Intercept) | 0.068 | | 0.047 | | 1.454 | | 0.189 | | Gaussian | |
|  | Na | 0.008 | | 0.003 | | 2.362 | | 0.050 | |  | |
|  | (Intercept) | -0.425 | | 0.295 | | -1.442 | | 0.192 | | Gaussian | |
|  | Hexp | 0.724 | | 0.355 | | 2.038 | | 0.081 | |  | |
|  | (Intercept) | 0.146 | | 0.010 | | 14.934 | | 1.45*10^-06^ | | Gaussian | |
|  | **Hd** | **0.053** | | **0.014** | | **3.722** | | **0.007** | |  | |
|  | (Intercept) | 0.151 | | 0.011 | | 13.739 | | 2.55*10^-06^ | | Gaussian | |
|  | **π** | **4.958** | | **1.776** | | **2.791** | | **0.027** | |  | |
|  | (Intercept) | 0.347 | | 0.366 | | 0.948 | | 0.375 | | Gaussian | |
|  | isoT | -0.414 | | 0.886 | | -0.468 | | 0.654 | |  | |
|  | (Intercept) | 0.276 | | 0.183 | | 1.510 | | 0.175 | | Linear | |
|  | maxTwarm12 | -0.003 | | 0.006 | | -0.553 | | 0.597 | |  | |
|  | (Intercept) | 0.102 | | 0.143 | | 0.712 | | 0.499 | | Linear | |
|  | Trange | 0.002 | | 0.004 | | 0.511 | | 0.625 | |  | |
|  | (Intercept) | 0.195 | | 0.024 | | 8.037 | | 8.85*10^-05^ | | Linear | |
|  | meanTwet4 | -0.003 | | 0.003 | | -0.917 | | 0.390 | |  | |
|  | (Intercept) | 0.141 | | 0.018 | | 8.008 | | 9.05*10^-05^ | | Gaussian | |
|  | Pwet12 | 3.761*10^-04^ | | 1.711*10^-04^ | | 2.199 | | 0.064 | |  | |
|  | (Intercept) | 0.179 | | 0.029 | | 6.233 | | 4.32*10^-04^ | | Linear | |
|  | Pdry12 | -0.001 | | 0.004 | | -0.172 | | 0.868 | |  | |
|  | (Intercept) | 0.150 | | 0.022 | | 6.837 | | 2.45*10^-04^ | | Exponential | |
|  | NDVI | 0.075 | | 0.060 | | 1.252 | | 0.251 | |  | |
|  | (Intercept) | 0.170 | | 0.012 | | 14.392 | | 1.86*10^-06^ | | Gaussian | |
|  | slope | 0.001 | | 0.001 | | 1.522 | | 0.172 | |  | |
|  | (Intercept) | 0.129 | | 0.012 | | 11.003 | | 1.14*10^-05^ | | Gaussian | |
|  | **Maxent** | **0.091** | | **0.020** | | **4.581** | | **0.003** | |  | |
| Shape | (Intercept) | 0.084 | | 0.029 | | 2.933 | | 0.022 | | Gaussian | |
|  | Na | 0.002 | | 0.002 | | 0.858 | | 0.419 | |  | |
|  | (Intercept) | -0.163 | | 0.095 | | -1.710 | | 0.131 | | Gaussian | |
|  | **Hexp** | **0.327** | | **0.115** | | **2.853** | | **0.025** | |  | |
|  | (Intercept) | 0.108 | | 0.007 | | 15.390 | | 1.18*10^-06^ | | Exponential | |
|  | Hd | 0.002 | | 0.010 | | 0.178 | | 0.863 | |  | |
|  | (Intercept) | 0.114 | | 0.005 | | 20.864 | | 1.46*10^-07^ | | Gaussian | |
|  | **π** | **-1.099** | | **0.391** | | **-2.812** | | **0.026** | |  | |
|  | (Intercept) | 0.151 | | 0.119 | | 1.263 | | 0.247 | | Gaussian | |
|  | isoT | -0.101 | | 0.287 | | -0.352 | | 0.735 | |  | |
|  | (Intercept) | 0.144 | | 0.068 | | 2.127 | | 0.071 | | Exponential | |
|  | maxTwarm12 | -0.001 | | 0.002 | | -0.518 | | 0.621 | |  | |
|  | (Intercept) | 0.139 | | 0.053 | | 2.628 | | 0.034 | | Exponential | |
|  | Trange | -0.001 | | 0.002 | | -0.577 | | 0.582 | |  | |
|  | (Intercept) | 0.111 | | 0.009 | | 12.887 | | 3.95*10^-06^ | | Exponential | |
|  | meanTwet4 | -3.165*10^-04^ | | 0.001 | | -0.260 | | 0.802 | |  | |
|  | (Intercept) | 0.111 | | 0.009 | | 11.936 | | 6.59*10^-06^ | | Gaussian | |
|  | Pwet12 | -2.590*10^-05^ | | 9.107*10^-05^ | | -0.284 | | 0.784 | |  | |
|  | (Intercept) | 0.106 | | 0.011 | | 10.010 | | 2.13*10^-05^ | | Gaussian | |
|  | Pdry12 | 3.418*10^-04^ | | 0.001 | | 0.232 | | 0.823 | |  | |
|  | (Intercept) | 0.091 | | 0.008 | | 11.259 | | 9.74*10^-06^ | | Linear | |
|  | **NDVI** | **0.054** | | **0.021** | | **2.534** | | **0.039** | |  | |
|  | (Intercept) | 0.107 | | 0.006 | | 17.551 | | 4.80*10^-07^ | | Exponential | |
|  | Slope | 2.252*10^-04^ | | 0.001 | | 0.280 | | 0.787 | |  | |
|  | (Intercept) | 0.109 | | 0.009 | | 11.559 | | 8.17*10^-06^ | | Gaussian | |
|  | Maxent | -0.001 | | 0.016 | | -0.071 | | 0.945 | |  | |
| GM | (Intercept) | 0.035 | | 0.007 | | 5.120 | | 0.001 | | Gaussian | |
|  | Na | -1.722*10^-04^ | | 4.898*10^-04^ | | -0.352 | | 0.735 | |  | |
|  | (Intercept) | 0.033 | | 0.035 | | 0.931 | | 0.383 | | Gaussian | |
|  | Hexp | -3.358*10^-04^ | | 0.043 | | -0.008 | | 0.994 | |  | |
|  | (Intercept) | 0.034 | | 0.002 | | 22.301 | | 9.22*10^-08^ | | Gaussian | |
|  | Hd | -0.002 | | 0.002 | | -0.916 | | 0.390 | |  | |
|  | (Intercept) | 0.034 | | 0.001 | | 26.182 | | 3.03*10^-08^ | | Gaussian | |
|  | π | -0.330 | | 0.211 | | -1.566 | | 0.161 | |  | |
|  | (Intercept) | 0.005 | | 0.025 | | 0.206 | | 0.842 | | Gaussian | |
|  | isoT | 0.066 | | 0.061 | | 1.076 | | 0.318 | |  | |
|  | (Intercept) | 0.049 | | 0.014 | | 3.405 | | 0.011 | | Gaussian | |
|  | maxTwarm12 | -5.417*10^-04^ | | 4.733*10^-04^ | | -1.145 | | 0.290 | |  | |
|  | (Intercept) | 0.037 | | 0.012 | | 3.055 | | 0.018 | | Gaussian | |
|  | Trange | -1.419*10^-04^ | | 3.634*10^-04^ | | -0.391 | | 0.708 | |  | |
|  | (Intercept) | 0.034 | | 0.002 | | 18.075 | | 3.92*10^-07^ | | Gaussian | |
|  | meanTwet4 | -2.204*10^-04^ | | 2.666*10^-04^ | | -0.827 | | 0.436 | |  | |
|  | (Intercept) | 0.033 | | 0.002 | | 15.895 | | 9.46*10^-07^ | | Gaussian | |
|  | Pwet12 | -9.332*10^-06^ | | 2.062*10^-05^ | | -0.453 | | 0.665 | |  | |
|  | (Intercept) | 0.032 | | 0.002 | | 13.304 | | 3.17*10^-06^ | | Gaussian | |
|  | Pdry12 | 3.439*10^-05^ | | 3.371*10^-04^ | | 0.102 | | 0.922 | |  | |
|  | (Intercept) | 0.033 | | 0.002 | | 14.195 | | 2.04*10^-06^ | | Gaussian | |
|  | NDVI | -4.025*10^-04^ | | 0.006 | | -0.064 | | 0.951 | |  | |
|  | (Intercept) | 0.033 | | 0.001 | | 23.457 | | 6.49*10^-08^ | | Gaussian | |
|  | Slope | -4.025*10^-05^ | | 1.838*10^-04^ | | -0.219 | | 0.833 | |  | |
|  | (Intercept) | 0.033 | | 0.002 | | 15.691 | | 1.03*10^-06^ | | Gaussian | |
|  | Maxent | -0.002 | | 0.004 | | -0.431 | | 0.679 | |  | |
| Scalation | (Intercept) | 0.182 | | 0.017 | | 10.919 | | 1.19*10^-05^ | | Gaussian | |
|  | Na | -0.003 | | 0.001 | | -2.178 | | 0.066 | |  | |
|  | (Intercept) | 0.049 | | 0.074 | | 0.653 | | 0.535 | | Linear | |
|  | Hexp | 0.119 | | 0.090 | | 1.329 | | 0.225 | |  | |
|  | (Intercept) | 0.151 | | 0.005 | | 33.487 | | 5.49*10^-09^ | | Gaussian | |
|  | **Hd** | **-0.008** | | **0.002** | | **-3.261** | | **0.014** | |  | |
|  | (Intercept) | 0.151 | | 0.005 | | 31.395 | | 8.59*10^-09^ | | Gaussian | |
|  | **π** | **-0.642** | | **0.175** | | **-3.662** | | **0.008** | |  | |
|  | (Intercept) | 0.065 | | 0.102 | | 0.634 | | 0.546 | | Exponential | |
|  | isoT | 0.198 | | 0.246 | | 0.803 | | 0.449 | |  | |
|  | (Intercept) | 0.194 | | 0.049 | | 3.977 | | 0.005 | | Linear | |
|  | maxTwarm12 | -0.002 | | 0.002 | | -0.965 | | 0.367 | |  | |
|  | (Intercept) | 0.069 | | 0.028 | | 2.432 | | 0.045 | | Linear | |
|  | **Trange** | **0.002** | | **0.001** | | **2.774** | | **0.028** | |  | |
|  | (Intercept) | 0.154 | | 0.007 | | 21.946 | | 1.03*10^-07^ | | Linear | |
|  | meanTwet4 | -0.001 | | 0.001 | | -1.214 | | 0.264 | |  | |
|  | (Intercept) | 0.141 | | 0.008 | | 17.979 | | 4.07*10^-07^ | | Linear | |
|  | Pwet12 | 6.580*10^-05^ | | 6.914*10^-05^ | | 0.952 | | 0.373 | |  | |
|  | (Intercept) | 0.134 | | 0.006 | | 22.102 | | 9.81*10^-08^ | | Linear | |
|  | Pdry12 | 0.002 | | 0.001 | | 2.283 | | 0.056 | |  | |
|  | (Intercept) | 0.140 | | 0.006 | | 22.227 | | 9.43*10^-08^ | | Linear | |
|  | NDVI | 0.022 | | 0.015 | | 1.498 | | 0.178 | |  | |
|  | (Intercept) | 0.150 | | 0.005 | | 29.994 | | 1.18*10^-08^ | | Gaussian | |
|  | **Slope** | **-0.001** | | **0.000** | | **-4.129** | | **0.004** | |  | |
|  | (Intercept) | 0.141 | | 0.007 | | 21.275 | | 1.27*10^-07^ | | Exponential | |
|  | Maxent | 0.011 | | 0.010 | | 1.035 | | 0.335 | |  | |

| **Table A9.3:** AIC statistics of GLS regression models applied to each response-predictor combination in *Podarcis bocagei* using different correlation structures for the error term to account for the effect of geographic structure in the data. | | | | | | | | | | |
| --- | --- | --- | --- | --- | --- | --- | --- | --- | --- | --- |
|  |  | **AIC** |  |  |  | **ΔAIC** |  |  |  | **max ΔAIC** |
| **Response** | **Predictor** | **Linear** | **Exponential** | **Gaussian** | **Rational Quadratic** | **Linear** | **Exponential** | **Gaussian** | **Rational Quadratic** |  |
| Size | Na | -40.32 | -39.53 | -40.76 | -40.19 | 0.45 | 1.23 | 0.00 | 0.58 | 1.23 |
|  | Hexp | -40.12 | -42.08 | -40.38 | -40.81 | 1.96 | 0.00 | 1.71 | 1.27 | 1.96 |
|  | Hd | -36.22 | -35.86 | -37.29 | -36.35 | 1.07 | 1.43 | 0.00 | 0.94 | 1.43 |
|  | π | -34.21 | -34.19 | -36.00 | -34.65 | 1.78 | 1.80 | 0.00 | 1.34 | 1.80 |
|  | isoT | -32.99 | -33.23 | -33.89 | -33.41 | 0.90 | 0.65 | 0.00 | 0.48 | 0.90 |
|  | maxTwarm12 | -39.93 | -39.88 | -39.93 | -39.86 | 0.00 | 0.05 | 3.62*10^-03^ | 0.07 | 0.07 |
|  | Trange | -33.78 | -34.03 | -34.00 | -33.86 | 0.25 | 0.00 | 0.03 | 0.17 | 0.25 |
|  | meanTwet4 | -32.76 | -33.04 | -33.41 | -33.06 | 0.65 | 0.37 | 0.00 | 0.35 | 0.65 |
|  | Pwet12 | -35.94 | -35.70 | -35.90 | -35.61 | 0.00 | 0.23 | 0.04 | 0.33 | 0.33 |
|  | Pdry12 | -36.74 | -35.58 | -35.49 | -35.51 | 0.00 | 1.16 | 1.25 | 1.23 | 1.25 |
|  | NDVI | -37.10 | -37.11 | -37.11 | -37.11 | 3.01*10^-03^ | 0.00 | 5.20*10^-05^ | 1.24*10^-03^ | 3.01*10^-03^ |
|  | Slope | -35.84 | -35.39 | -35.89 | -35.50 | 0.05 | 0.50 | 0.00 | 0.40 | 0.50 |
|  | Maxent | -32.82 | -33.10 | -33.92 | -33.34 | 1.10 | 0.82 | 0.00 | 0.58 | 1.10 |
| Shape | Na | -46.10 | -46.02 | -46.06 | -46.01 | 0.00 | 0.07 | 0.04 | 0.08 | 0.08 |
|  | Hexp | -50.65 | -50.30 | -50.44 | -50.29 | 0.00 | 0.35 | 0.21 | 0.36 | 0.36 |
|  | Hd | -44.45 | -44.20 | -44.60 | -44.08 | 0.15 | 0.41 | 0.00 | 0.52 | 0.52 |
|  | π | -46.55 | -46.82 | -47.23 | -46.70 | 0.68 | 0.40 | 0.00 | 0.52 | 0.68 |
|  | isoT | -45.33 | -44.88 | -45.23 | -44.78 | 0.00 | 0.44 | 0.09 | 0.54 | 0.54 |
|  | maxTwarm12 | -45.70 | -45.34 | -45.65 | -45.30 | 0.00 | 0.35 | 0.05 | 0.40 | 0.40 |
|  | Trange | -45.30 | -44.95 | -45.24 | -44.88 | 0.00 | 0.35 | 0.05 | 0.41 | 0.41 |
|  | meanTwet4 | -46.85 | -46.39 | -46.78 | -46.32 | 0.00 | 0.46 | 0.07 | 0.53 | 0.53 |
|  | Pwet12 | -44.71 | -44.40 | -44.75 | -44.33 | 0.04 | 0.35 | 0.00 | 0.42 | 0.42 |
|  | Pdry12 | -48.03 | -47.40 | -47.81 | -47.32 | 0.00 | 0.62 | 0.21 | 0.70 | 0.70 |
|  | NDVI | -48.07 | -47.66 | -48.38 | -47.70 | 0.31 | 0.72 | 0.00 | 0.69 | 0.72 |
|  | Slope | -44.44 | -44.21 | -44.66 | -44.10 | 0.22 | 0.45 | 0.00 | 0.56 | 0.56 |
|  | Maxent | -45.69 | -45.26 | -45.59 | -45.17 | 0.00 | 0.42 | 0.10 | 0.52 | 0.52 |
| GM | Na | -95.60 | -95.47 | -95.62 | -95.46 | 0.02 | 0.15 | 0.00 | 0.15 | 0.15 |
|  | Hexp | -94.41 | -94.34 | -94.57 | -94.42 | 0.16 | 0.24 | 0.00 | 0.15 | 0.24 |
|  | Hd | -93.20 | -93.29 | -93.55 | -93.43 | 0.35 | 0.26 | 0.00 | 0.12 | 0.35 |
|  | π | -94.10 | -94.10 | -94.50 | -94.35 | 0.40 | 0.40 | 0.00 | 0.16 | 0.40 |
|  | isoT | -105.60 | -105.16 | -105.31 | -105.15 | 0.00 | 0.44 | 0.29 | 0.45 | 0.45 |
|  | maxTwarm12 | -94.45 | -93.65 | -93.69 | -93.69 | 0.00 | 0.80 | 0.75 | 0.76 | 0.80 |
|  | Trange | -95.85 | -95.47 | -95.79 | -95.50 | 0.00 | 0.38 | 0.06 | 0.35 | 0.38 |
|  | meanTwet4 | -99.13 | -98.79 | -98.91 | -98.78 | 0.00 | 0.34 | 0.23 | 0.35 | 0.35 |
|  | Pwet12 | -93.23 | -93.95 | -93.90 | -93.97 | 0.74 | 0.02 | 0.07 | 0.00 | 0.74 |
|  | Pdry12 | -100.16 | -99.78 | -99.96 | -99.77 | 0.00 | 0.37 | 0.20 | 0.39 | 0.39 |
|  | NDVI | -93.65 | -93.59 | -93.92 | -93.77 | 0.27 | 0.33 | 0.00 | 0.15 | 0.33 |
|  | Slope | -93.43 | -93.53 | -93.80 | -93.68 | 0.38 | 0.27 | 0.00 | 0.13 | 0.38 |
|  | Maxent | -101.08 | -101.08 | -101.08 | -101.08 | 0.01 | 2.22*10^-03^ | 0.00 | 4.12*10^-03^ | 0.01 |
| Scalation | Na | -56.15 | -56.15 | -56.15 | -56.15 | 3.63*10^-03^ | 1.41*10^-04^ | 0.00 | 3.95*10^-04^ | 3.63*10^-03^ |
|  | Hexp | -54.74 | -54.74 | -54.74 | -54.74 | 1.37*10^-03^ | 2.02*10^-04^ | 0.00 | 1.42*10^-03^ | 1.42*10^-03^ |
|  | Hd | -57.09 | -57.66 | -57.09 | -57.06 | 0.57 | 0.00 | 0.57 | 0.60 | 0.60 |
|  | π | -56.58 | -56.58 | -56.58 | -56.58 | 8.15*10^-04^ | 0.00 | 4.21*10^-05^ | 4.09*10^-04^ | 8.15*10^-04^ |
|  | isoT | -56.44 | -56.45 | -56.45 | -56.44 | 1.74*10^-03^ | 2.35*10^-04^ | 0.00 | 1.51*10-03 | 1.74*10^-03^ |
|  | maxTwarm12 | -54.23 | -54.24 | -54.24 | -54.23 | 1.29*10^-03^ | 1.38*10^-04^ | 0.00 | 1.54*10-03 | 1.54*10^-03^ |
|  | Trange | -54.31 | -54.31 | -54.31 | -54.31 | 1.36*10^-03^ | 1.81*10^-04^ | 0.00 | 1.47*10-03 | 1.47*10^-03^ |
|  | meanTwet4 | -54.47 | -54.47 | -54.47 | -54.47 | 1.53*10^-03^ | 2.00*10^-04^ | 0.00 | 1.48*10-03 | 1.53*10^-03^ |
|  | Pwet12 | -57.76 | -57.76 | -57.76 | -57.76 | 2.26*10^-03^ | 1.84*10^-04^ | 0.00 | 6.88*10^-04^ | 2.26*10^-03^ |
|  | Pdry12 | -54.87 | -54.87 | -54.87 | -54.87 | 1.62*10^-03^ | 2.28*10^-04^ | 0.00 | 1.44*10^-03^ | 1.62*10^-03^ |
|  | NDVI | -54.23 | -54.23 | -54.23 | -54.23 | 1.34*10^-03^ | 1.76*10^-04^ | 0.00 | 1.46*10^-03^ | 1.46*10^-03^ |
|  | Slope | -59.69 | -59.69 | -59.69 | -59.69 | 2.13*10^-03^ | 2.31*10^-04^ | 0.00 | 1.13*10^-03^ | 2.13*10^-03^ |
|  | Maxent | -55.81 | -55.81 | -55.81 | -55.81 | 1.44*10^-03^ | 2.02*10^-04^ | 0.00 | 1.83*10^-03^ | 1.83*10^-03^ |

| **Table A9.4:** AIC statistics of GLS regression models applied to each response-predictor combination in *Podarcis vaucheri* using different correlation structures for the error term to account for the effect of geographic structure in the data. | | | | | | | | | | |
| --- | --- | --- | --- | --- | --- | --- | --- | --- | --- | --- |
|  |  | **AIC** |  |  |  | **ΔAIC** |  |  |  | **max ΔAIC** |
| **Response** | **Predictor** | **Linear** | **Exponential** | **Gaussian** | **Rational Quadratic** | **Linear** | **Exponential** | **Gaussian** | **Rational Quadratic** |  |
| Size | Na | -36.20 | -35.32 | -37.07 | -36.19 | 0.87 | 1.75 | 0.00 | 0.88 | 1.75 |
|  | Hexp | -36.20 | -36.20 | -36.20 | -36.20 | 7.04E-04 | 5.60E-05 | 0.00 | 3.74E-04 | 7.04*10^-4^ |
|  | Hd | -41.83 | -41.83 | -41.83 | -41.83 | 6.33E-04 | 6.00E-05 | 0.00 | 3.25E-04 | 6.33*10^-4^ |
|  | π | -38.74 | -38.74 | -38.74 | -38.74 | 8.20E-04 | 2.40E-04 | 0.00 | 3.40E-04 | 8.20*10^-4^ |
|  | isoT | -32.39 | -32.60 | -32.98 | -32.65 | 0.58 | 0.38 | 0.00 | 0.32 | 0.58 |
|  | maxTwarm12 | -33.44 | -32.95 | -33.06 | -32.96 | 0.00 | 0.49 | 0.37 | 0.48 | 0.49 |
|  | Trange | -33.38 | -32.57 | -32.87 | -32.64 | 0.00 | 0.81 | 0.52 | 0.74 | 0.81 |
|  | meanTwet4 | -34.07 | -33.76 | -33.66 | -33.64 | 0.00 | 0.32 | 0.42 | 0.43 | 0.43 |
|  | Pwet12 | -37.00 | -37.02 | -37.09 | -37.00 | 0.09 | 0.07 | 0.00 | 0.08 | 0.09 |
|  | Pdry12 | -33.09 | -32.34 | -32.88 | -32.42 | 0.00 | 0.75 | 0.21 | 0.67 | 0.75 |
|  | NDVI | -33.83 | -33.83 | -33.83 | -33.83 | 7.36E-04 | 0.00 | 5.66E-10 | 4.55E-04 | 7.36*10^-4^ |
|  | Slope | -34.03 | -32.76 | -34.64 | -33.54 | 0.61 | 1.87 | 0.00 | 1.10 | 1.87 |
|  | Maxent | -45.04 | -44.99 | -45.13 | -45.00 | 0.09 | 0.14 | 0.00 | 0.13 | 0.14 |
| Shape | Na | -48.70 | -48.70 | -48.70 | -48.70 | 2.49E-03 | 2.77E-04 | 0.00 | 3.21E-04 | 2.49*10^-3^ |
|  | Hexp | -48.36 | -48.58 | -49.45 | -48.57 | 1.09 | 0.87 | 0.00 | 0.87 | 1.09 |
|  | Hd | -47.84 | -47.84 | -47.84 | -47.84 | 1.61E-03 | 0.00 | 0.00 | 7.16E-04 | 1.61*10^-3^ |
|  | π | -47.90 | -48.00 | -49.30 | -48.36 | 1.40 | 1.30 | 0.00 | 0.94 | 1.40 |
|  | isoT | -47.96 | -47.96 | -47.96 | -47.96 | 7.41E-04 | 2.67E-05 | 0.00 | 1.01E-03 | 1.01*10^-3^ |
|  | maxTwarm12 | -48.14 | -48.14 | -48.14 | -48.14 | 8.72E-04 | 0.00 | 0.00 | 9.11E-04 | 9.11*10^-4^ |
|  | Trange | -48.22 | -48.22 | -48.22 | -48.22 | 1.18E-03 | 0.00 | 2.16E-04 | 1.21E-03 | 1.21*10^-3^ |
|  | meanTwet4 | -47.89 | -47.89 | -47.89 | -47.89 | 1.15E-03 | 0.00 | 0.00 | 9.60E-04 | 1.15*10^-3^ |
|  | Pwet12 | -47.90 | -47.91 | -47.91 | -47.90 | 1.31E-03 | 9.40E-05 | 0.00 | 1.14E-03 | 1.31*10^-3^ |
|  | Pdry12 | -47.87 | -47.87 | -47.87 | -47.87 | 1.13E-03 | 8.35E-08 | 0.00 | 9.03E-04 | 1.13*10^-3^ |
|  | NDVI | -52.24 | -51.78 | -51.66 | -51.62 | 0.00 | 0.46 | 0.58 | 0.61 | 0.61 |
|  | Slope | -47.90 | -47.90 | -47.90 | -47.90 | 2.14E-03 | 0.00E+00 | 0.00 | 8.95E-09 | 2.14*10^-3^ |
|  | Maxent | -47.81 | -47.81 | -47.81 | -47.81 | 1.19E-03 | 2.15E-07 | 0.00 | 1.03E-03 | 1.19*10^-3^ |
| Scalation | Na | -56.98 | -56.52 | -57.40 | -56.88 | 0.41 | 0.88 | 0.00 | 0.52 | 0.88 |
|  | Hexp | -56.50 | -54.79 | -53.80 | -55.32 | 0.00 | 1.71 | 2.70 | 1.18 | 2.70 |
|  | Hd | -56.15 | -55.48 | -56.73 | -56.41 | 0.58 | 1.26 | 0.00 | 0.32 | 1.26 |
|  | π | -56.33 | -55.46 | -57.08 | -56.73 | 0.75 | 1.62 | 0.00 | 0.35 | 1.62 |
|  | isoT | -54.80 | -55.02 | -54.85 | -54.88 | 0.22 | 0.00 | 0.17 | 0.14 | 0.22 |
|  | maxTwarm12 | -55.60 | -55.03 | -53.97 | -54.26 | 0.00 | 0.56 | 1.62 | 1.34 | 1.62 |
|  | Trange | -60.16 | -60.15 | -60.16 | -60.11 | 0.00 | 1.34E-02 | 8.41E-08 | 5.28E-02 | 5.28*10^-2^ |
|  | meanTwet4 | -56.19 | -55.43 | -54.49 | -54.75 | 0.00 | 0.76 | 1.70 | 1.44 | 1.70 |
|  | Pwet12 | -55.57 | -54.90 | -53.75 | -54.01 | 0.00 | 0.67 | 1.82 | 1.56 | 1.82 |
|  | Pdry12 | -58.75 | -58.67 | -58.75 | -58.66 | 0.00 | 0.08 | 0.00 | 0.10 | 0.10 |
|  | NDVI | -56.67 | -55.94 | -53.75 | -55.11 | 0.00 | 0.73 | 2.92 | 1.56 | 2.92 |
|  | Slope | -56.91 | -56.17 | -57.82 | -57.75 | 0.90 | 1.64 | 0.00 | 0.06 | 1.64 |
|  | Maxent | -54.67 | -55.72 | -54.67 | -54.96 | 1.05 | 0.00 | 1.05 | 0.76 | 1.05 |
| GM | Na | -74.54 | -74.54 | -74.54 | -74.54 | 2.11E-03 | 2.25E-04 | 0.00 | 9.26E-04 | 2.11*10^-3^ |
|  | Hexp | -74.38 | -74.38 | -74.39 | -74.38 | 2.08E-03 | 2.32E-04 | 0.00 | 1.14E-03 | 2.08*10^-3^ |
|  | Hd | -75.40 | -75.40 | -75.40 | -75.40 | 1.55E-03 | 2.26E-04 | 0.00 | 1.88E-03 | 1.88*10^-3^ |
|  | π | -77.09 | -77.09 | -77.09 | -77.09 | 2.43E-03 | 3.21E-05 | 0.00 | 1.41E-03 | 2.43*10^-3^ |
|  | isoT | -75.76 | -75.76 | -75.76 | -75.76 | 2.86E-03 | 1.71E-04 | 0.00 | 5.32E-04 | 2.86*10^-3^ |
|  | maxTwarm12 | -75.93 | -75.93 | -75.93 | -75.93 | 2.71E-03 | 1.89E-04 | 0.00 | 6.07E-04 | 2.71*10^-3^ |
|  | Trange | -74.58 | -74.58 | -74.58 | -74.58 | 2.13E-03 | 2.01E-04 | 0.00 | 5.56E-04 | 2.13*10^-3^ |
|  | meanTwet4 | -75.22 | -75.22 | -75.22 | -75.22 | 2.58E-03 | 1.97E-04 | 0.00 | 6.50E-04 | 2.58*10^-3^ |
|  | Pwet12 | -74.64 | -74.64 | -74.64 | -74.64 | 2.30E-03 | 2.00E-04 | 0.00 | 5.91E-04 | 2.30*10^-3^ |
|  | Pdry12 | -74.40 | -74.40 | -74.40 | -74.40 | 2.21E-03 | 2.09E-04 | 0.00 | 6.68E-04 | 2.21*10^-3^ |
|  | NDVI | -74.39 | -74.39 | -74.39 | -74.39 | 2.20E-03 | 2.17E-04 | 0.00 | 7.64E-04 | 2.20*10^-3^ |
|  | Slope | -74.45 | -74.45 | -74.45 | -74.45 | 1.34E-03 | 5.10E-05 | 0.00 | 4.45E-04 | 1.34*10^-3^ |
|  | Maxent | -74.62 | -74.62 | -74.62 | -74.62 | 2.06E-03 | 2.12E-04 | 0.00 | 6.51E-04 | 2.06*10^-3^ |
