# Supplementary material for: Where does diversity come from? Linking geographical patterns of morphological, genetic, and environmental variation in wall lizards
Source: BMC Evol Biol. 2018 Aug 22;18:124. doi: 10.1186/s12862-018-1237-7 (PMC6113677; doi:10.1186/s12862-018-1237-7)
Supplement: Supplementary file 3 — Geographic coordinates in the WGS1984 system, number of individuals sampled per sex (Nm: males, Nf: females) and in total (Ntot), and abbreviation (CODE) for the examined populations. (DOCX 16 kb) [file 12862_2018_1237_MOESM3_ESM.docx]

**Additional file 1:** Geographic coordinates in the WGS1984 system, number of individuals sampled per sex (Nm: males, Nf: females) and in total (Ntot), and abbreviation (CODE) for the examined populations.

|  |  | **Latitude** | **Longitude** | **Nm** | **Nf** | **Ntot** | **CODE** |
| --- | --- | --- | --- | --- | --- | --- | --- |
| ***P. bocagei*** | Castro Laboreiro | 41˚ 59.69' N | 9˚ 45.24' W | 20 | 11 | **31** | CLAB |
|  | Gerês | 41˚ 46.94' N | 9˚ 51.29' W | 17 | 17 | **34** | GERES |
|  | Gião | 41˚ 18.78' N | 9˚ 18.5' W | 20 | 16 | **36** | GIAO |
|  | Madalena | 41˚ 6.24' N | 9˚ 20.32' W | 17 | 18 | **35** | MDLN |
|  | Montesinho | 41˚ 58.76' N | 7˚ 12.28' W | 19 | 16 | **35** | MNTS |
|  | Moledo | 41˚ 50.31' N | 9˚ 7.56' W | 18 | 10 | **28** | MOL |
|  | São Mamede do Coronado | 41˚ 17.12' N | 9˚ 25.53' W | 18 | 18 | **36** | SMDC |
|  | Subportela | 41˚ 41.25' N | 9˚ 16.91' W | 18 | 18 | **36** | SUBP |
|  | Vila Pouca de Aguiar | 41˚ 26.75' N | 8˚ 19.67' W | 19 | 15 | **34** | VPAG |
| ***P. vaucheri*** | Bab Taza | 35˚ 1.35' N | 6˚ 47.73' W | 20 | 20 | **40** | BTAZ |
|  | Debdou | 33˚ 52.35' N | 4˚ 57.67' W | 19 | 18 | **37** | DEBD |
|  | Imlil | 31˚ 6.04' N | 8˚ 5.13' W | 13 | 11 | **24** | IML |
|  | Ketama | 34˚ 52.69' N | 5˚ 23.35' W | 12 | 14 | **26** | KTM |
|  | Midelt | 32˚ 40.94' N | 5˚ 15.44' W | 17 | 16 | **33** | MDLT |
|  | Mischliffen | 33˚ 24.33' N | 6˚ 53.8' W | 14 | 11 | **25** | MSCL |
|  | Oukaimeden | 31˚ 12.21' N | 8˚ 8.3' W | 16 | 12 | **28** | OUKM |
|  | Tazeka NP | 34˚ 6.25' N | 5˚ 55.65' W | 17 | 17 | **34** | TAZA |
|  | Tislit Lake | 32˚ 11.78' N | 6˚ 21.42' W | 19 | 19 | **38** | TISL |
