# Supplementary material for: Where does diversity come from? Linking geographical patterns of morphological, genetic, and environmental variation in wall lizards
Source: BMC Evol Biol. 2018 Aug 22;18:124. doi: 10.1186/s12862-018-1237-7 (PMC6113677; doi:10.1186/s12862-018-1237-7)
Supplement: Supplementary file 4 — Geometric morphometric procedures for quantifying variation in dorsal head shape, provides a detailed description of all steps involved in obtaining head shape data, including landmark definitions and digitizing, superimposition of landmark coordinates and other pre-processing applied to GM data. (DOCX 13 kb) [file 12862_2018_1237_MOESM4_ESM.docx]

**Additional file 2:** Geometric Morphometric procedures for quantifying variation in dorsal head shape

To quantify variation in dorsal head shape, we used tpsDig (Rohlf, 2015) to record the Cartesian coordinates of 24 landmarks, located at the intersections of the scales covering the pileus of lacertid lizards (Fig. 1E). These scales are structurally linked to underlying skull elements (Costantini et al. 2009) and the landmarks recorded have been previously used for quantifying variation within (Lazić et al. 2015) and across (Kaliontzopoulou et al. 2008, 2010a, 2012b) individuals of wall lizards. All landmarks were digitized by the same person (AK) to reduce measurement error. Based on the recorded landmark coordinates, we then used the function gpagen of the geomorph R-package (Adams et al. 2016) to perform a Generalized Procrustes Analysis (Rohlf and Slice 1990) which removed the effects of scale, position and orientation and provided variables for shape analyses.

Because the landmark configuration included 10 pairs of bilaterally symmetrical landmarks, and here we were not interested in studying asymmetry patterns, we removed all asymmetry effects from landmark configurations. For this purpose, we reflected the landmark coordinates of each individual across the midline of the landmark configuration to obtain mirror images, which were then superimposed together with the original data. Then, the symmetric shape component for each individual was obtained by averaging the original and reflected data, and used to investigate divergence among and diversity within populations in dorsal head shape. Because the head of lacertids is known to exhibit both sexual dimorphism and allometric variation (i.e. Kaliontzopoulou et al. 2008), we standardized these effects by fitting a linear model with sex and centroid size as predictors and retaining the matrix of residuals as sex mean-centered, size-corrected shape variables.

**References not included in the main text**

Adams, D. C., M.L. Collyer, A. Kaliontzopoulou and E. Sherratt. 2016. geomorph: Geometric Morphometric Analyses of 2D/3D Landmark Data. R package version 3.0.3. Available at: <https://cran.r-project.org/web/packages/geomorph/index.html>

Costantini, D., Lapresa Alonso, M., Moazen, M., Bruner, E. 2009. The relationship between cephalic scales and bones in lizards: A preliminary microtomographic survey on three lacertid species. The Anatomical Record, 293, 183-194.

Rohlf, F.J. 2015. tpsDig2 v. 2.22. Ecology and Evolution, SUNY at Stony Brook.

Rohlf, F. J., and D. E. Slice. 1990. Extensions of the Procrustes method for the optimal superimposition of landmarks. Syst. Zool. 39:40–59.
