# Supplementary material for: Where does diversity come from? Linking geographical patterns of morphological, genetic, and environmental variation in wall lizards
Source: BMC Evol Biol. 2018 Aug 22;18:124. doi: 10.1186/s12862-018-1237-7 (PMC6113677; doi:10.1186/s12862-018-1237-7)
Supplement: Supplementary file 7 — Genetic analyses, provides a detailed account of of laboratory and analytical procedures followed to obtain genetic data, and the corresponding descriptors of population diversity and differentiation. (DOCX 81 kb) [file 12862_2018_1237_MOESM7_ESM.docx]

**Additional file 5:** Genetic analyses

**5.1 Methods**

**5.1.1 Microsatellite loci**

**5.1.1.1 Genotyping**

DNA was extracted following standard protocols (Sambrook *et al.* 1989) from tail muscle belonging exactly to the same individuals included in the morphological analyses (305 *P bocagei* and 284 *P. vaucheri;* see Additional file 1).

After preliminary essays to evaluate amplification conditions and observe microsatellite length range, the nine markers were arranged into two different multiplex PCR reactions. These were carried out with three primers per locus, following the M13-tailed primer method (Oetting et al. 1995). In brief, this method consists in adding four different universal M13 primers labeled with fluorescent dyes (6-FAM, VIC, NED and PET) which anneal to tails incorporated 5’ into the forward primer sequence. The list of the markers used, as well as their organization into multiplexes and composition of each multiplex primer mix is given in Table 5.1.

Multiplex PCR reactions were carried out in 10 μL volumes containing 5 μL of Master Mix (Quiagen), 3 μL of H2O, 1 μL of preprepared primer mix and 1 μL of DNA. Both multiplexes were amplified following a touchdown PCR procedure with the same general structure, differing only in annealing temperatures. For both multiplexes PCR started by an initial denaturing step of 15’ at 95ºC. This was followed by 40 cycles of 95ºC for 30’’, 30’’ of annealing (see temperatures below) and 20’’ of extension at 72ºC. This was followed by a final 30’ at 60ºC. For multiplex 1, annealing temperature started at 58ºC and dropped 0.3ºC per cycle for the first 10 cycles; subsequently, the temperature remained stable at 55ºC for the remaining 30 cycles. For multiplex 2, annealing started at 60ºC and was lowered by a rate of 0.5ºC per cycle for the first 15 cycles and was kept constant at 53ºC for the subsequent 25 cycles.

| **Table 5.1.** Microsatellite loci used and multiplexing conditions; volumes below reflect the composition of the primer mix used in each multiplex PCR reaction. Loci are described in Pinho *et al.* 2004. | | | | | | | | | | | | |
| --- | --- | --- | --- | --- | --- | --- | --- | --- | --- | --- | --- | --- |
| Locus | Multiplex | Dye | Primer mix composition (µL) | | | | | | | | | |
|  |  |  | Forward primer (10µM)^1^ | Reverse primer (100µM) | FAM | VIC | NED | | PET | | H_2_O | |
| Pb10 | 1 | FAM | 0.4 | 0.4 | 0.4 | 1 | 0.4 | | 2 | | 38.6 | |
| Pb37 | 1 | VIC | 1 | 1 |  |  |  |  |  |  |  |  |
| Pb11 | 1 | NED | 0.4 | 0.4 |  |  |  |  |  |  |  |  |
| Pb50 | 1 | PET | 2 | 2 |  |  |  |  |  |  |  |  |
| Pb20 | 2 | FAM | 0.4 | 0.4 | 1 | 1 | 0.4 | | 2 | | 36.8 | |
| Pb47 | 2 | FAM | 0.6 | 0.6 |  |  |  |  |  |  |  |  |
| Pb55 | 2 | VIC | 1 | 1 |  |  |  |  |  |  |  |  |
| Pb73 | 2 | NED | 0.4 | 0.4 |  |  |  |  |  |  |  |  |
| Pb66 | 2 | PET | 0.2 | 0.2 |  |  |  |  |  |  |  |  |
| ^1^A M13 tail was added 5' to the published primer sequence. | | | | | | | |  | |  | |  |

PCR products were separated in ABI Prism® 3130xl Genetic Analyzer (Applied Biosystems) along with an internal size standard (Genescan-500 LIZ, ABI). Alleles were scored based on sizing bin windows using GeneMapper® software v3.1.2 (Applied Biosystems).

**5.1.1.2 Analytical procedures**

After genotyping, the data were imported into Microchecker 2.2.3 (Van Oosterhout et al. 2004) to evaluate the presence of genotyping errors such as null alleles. We used 10000 replicates per locus per population and Bonferroni correction throughout the analyses. Because several loci were inferred to systematically present null alleles across populations in both species (see Results) we opted by using an approach that explicitly takes into account the presence of null alleles in several of the downstream analyses.

We opted by the method outlined by Chapuis and Estoup (2007) and implemented in software FreeNA (available in <http://www1.montpellier.inra.fr/CBGP/software/FreeNA/>), to i) estimate allele frequencies corrected for the existence of null alleles; ii) calculate Fst (Weir 1996) based on corrected data sets; iii) estimate the Cavalli-Sforza and Edwards (1967) chord distance also accounting for null alleles. This method requires distinguishing between genotypes that are missing because they represent homozygotes for null alleles and those that are missing for other reasons (e.g. low quality or degraded samples, other technical problems). To assist us on this distinction, we used the following rationale: samples that have missing data at many loci are likely degraded samples that fail to amplify for reasons other than those that cause null alleles. Samples that have missing data sporadically at only a few loci are more likely to be homozygous for null alleles at those loci. To acknowledge a sample as “failed”, we considered a threshold of more than 3 missing loci for *P. bocagei* and of 5 missing loci for *P. vaucheri,* which correspond to the 95 percentile of missing data in both cases. This strategy allowed us to be consistent among species, since the markers were developed for *P. bocagei* and, for this reason, it is likely that *P. vaucheri* carries more null alleles. This implied classifying as failed due to other technical problems 13 samples out of 305 in *P. bocagei* and 7 samples out of 284 *P. vaucheri* samples. After manually correcting missing genotypes according to this classification, data were imported into FreeNA. Although FreeNA incorporates bootstrap resampling among loci to determine confidence values on Fst (we used 1000 bootstrap replicates in our main analyses), it does not allow performing permutations of individuals among populations to determine the significance of Fst values. Therefore, for each species we generated 100 permuted datasets that were subsequently ran in FreeNA for comparison.

Besides genetic distances and Fst to investigate differences among populations, we were also interested in measures of diversity to evaluate within population variability. For this purpose, we calculated the number of alleles per population and expected heterozygosity. For the first measure we did not account for null alleles because we cannot evaluate in a satisfactory manner how many alleles are actually “hidden” behind a null allele. Sample sizes are expected to have a strong influence on the number of alleles detected in a population. To minimize the problem of differential sample sizes among populations, we used a resampling approach as outlined in Salvi *et al.* (2013): for each population with sample size greater than the minimum sample size across populations (s), we produced 10000 resampled datasets including s individuals, and took the mean of the number of alleles across those samples.

In the calculations of expected heterozygosity, we took into account allele frequencies corrected for null alleles as outputted by FreeNA.

With the exceptions noted (Microchecker and FreeNA), all calculations, format conversions and resampling or permutation of datasets were conducted using scripts written in Python 2.7.X (available from the authors upon request).

**5.1.2 Mitochondrial DNA**

**5.1.2.1 Laboratory procedures**

Sequences from individuals from the same localities under investigation have been published for both *P. bocagei*  and *P. vaucheri* (Pinho *et al.* 2007b, Kaliontzopoulou *et al.* 2011). We used these sequences along with newly sequenced individuals to build datasets including 5-10 individuals per locality. Table S5.2 summarizes this information and presents accession numbers.

We used primers ND4 and Leu (Arévalo *et al.* 1994) to amplify a portion of the NADH dehydrogenase subunit 4 (ND4) gene and adjacent tRNAs. PCRs were carried out in 10 μL volumes containing 5 μL of Mytaq Mastermix, 3.5 μL of H2O, 0.5 μL of each primer at 10 μM and 0.5 μL of DNA (concentration not quantified). The PCR cycle was as follows: initial denaturing step at 95ºC for 15 minutes, followed by 40 cycles of 95ºC for 40’’, 54ºC for 45’’ and 72ºC for 45’’. This was followed by a final step at 60ºC for 12’.

Cleaning and sequencing of PCR products (using primer HisR; Pinho *et al.* 2006) was carried out by Macrogen Inc.

**Table 5.2.** Individuals included in the analyses of mitochondrial DNA. See Supplementary Table 1 for locality information.

| Species | Locality acronym | Sample | Source | Accession number |
| --- | --- | --- | --- | --- |
| *P. bocagei* | SUBPOR | 3.221 | this study | KY461777 |
| *P. bocagei* | SUBPOR | 3.224 | this study | KY461784 |
| *P. bocagei* | SUBPOR | 3.225 | this study | KY461783 |
| *P. bocagei* | SUBPOR | 3.227 | this study | KY461782 |
| *P. bocagei* | SUBPOR | 3.229 | this study | KY461781 |
| *P. bocagei* | SUBPOR | 3.239 | this study | KY461780 |
| *P. bocagei* | SUBPOR | 3.246 | this study | KY461779 |
| *P. bocagei* | SUBPOR | 3.247 | this study | KY461778 |
| *P. bocagei* | GERES | 3.330 | this study | KY461786 |
| *P. bocagei* | GERES | 3.338 | this study | KY461787 |
| *P. bocagei* | GERES | 3.341 | this study | KY461785 |
| *P. bocagei* | GERES | 3.343 | this study | KY461788 |
| *P. bocagei* | GERES | 3.349 | this study | KY461789 |
| *P. bocagei* | GERES | 3.350 | this study | KY461790 |
| *P. bocagei* | GERES | 3.361 | this study | KY461791 |
| *P. bocagei* | MDLN | 3.98 | this study | KY461794 |
| *P. bocagei* | MDLN | 3.104 | this study | KY461795 |
| *P. bocagei* | MDLN | 3.110 | this study | KY461793 |
| *P. bocagei* | MDLN | 3.120 | this study | KY461792 |
| *P. bocagei* | MDLN | DB8029 | Pinho et al. 2007b | EF081133 |
| *P. bocagei* | MDLN | DB8031 | Pinho et al. 2007b | EF081116 |
| *P. bocagei* | MDLN | DB8032 | Pinho et al. 2007b | EF081116 |
| *P. bocagei* | MDLN | DB8165 | Pinho et al. 2007b | EF081133 |
| *P. bocagei* | MDLN | DB8168 | Pinho et al. 2007b | EF081116 |
| *P. bocagei* | MDLN | DB8171 | Pinho et al. 2007b | EF081116 |
| *P. bocagei* | MDLN | DB8173 | Pinho et al. 2007b | EF081116 |
| *P. bocagei* | MDLN | DB8178 | Pinho et al. 2007b | EF081116 |
| *P. bocagei* | GIAO | 3.52 | this study | KY461796 |
| *P. bocagei* | GIAO | 3.56 | this study | KY461797 |
| *P. bocagei* | GIAO | 3.57 | this study | KY461798 |
| *P. bocagei* | GIAO | 3.60 | this study | KY461799 |
| *P. bocagei* | GIAO | 3.61 | this study | KY461800 |
| *P. bocagei* | GIAO | 3.63 | this study | KY461801 |
| *P. bocagei* | GIAO | DB8012 | Pinho et al. 2007b | EF081116 |
| *P. bocagei* | GIAO | DB9631 | Pinho et al. 2007b | EF081116 |
| *P. bocagei* | CLAB | 3.298 | this study | KY461802 |
| *P. bocagei* | CLAB | 3.301 | this study | KY461803 |
| *P. bocagei* | CLAB | 3.303 | this study | KY461804 |
| *P. bocagei* | CLAB | 3.306 | this study | KY461805 |
| *P. bocagei* | CLAB | 3.309 | this study | KY461806 |
| *P. bocagei* | CLAB | 3.315 | this study | KY461807 |
| *P. bocagei* | CLAB | 3.321 | this study | KY461808 |
| **Table 5.2.** (continued) | | | | |
| Species | Locality acronym | Sample | Source | Accession number |
| *P. bocagei* | MNTS | 3.166 | this study | KY461809 |
| *P. bocagei* | MNTS | 3.173 | this study | KY461810 |
| *P. bocagei* | MNTS | 3.266 | this study | KY461811 |
| *P. bocagei* | MNTS | 3.267 | this study | KY461812 |
| *P. bocagei* | MNTS | 3.268 | this study | KY461813 |
| *P. bocagei* | MNTS | DB8056 | Pinho et al. 2007b | EF081117 |
| *P. bocagei* | MNTS | DB8060 | Pinho et al. 2007b | EF081117 |
| *P. bocagei* | MNTS | DB8062 | Pinho et al. 2007b | EF081117 |
| *P. bocagei* | MNTS | DB8065 | Pinho et al. 2007b | EF081118 |
| *P. bocagei* | MNTS | DB8069 | Pinho et al. 2007b | EF081118 |
| *P. bocagei* | MNTS | DB8072 | Pinho et al. 2007b | EF081117 |
| *P. bocagei* | MNTS | DB8103 | Pinho et al. 2007b | EF081117 |
| *P. bocagei* | MOLEDO | 3.3 | Pinho et al. 2007b | EF081120 |
| *P. bocagei* | MOLEDO | 3.18 | this study | KY461814 |
| *P. bocagei* | MOLEDO | 3.34 | Pinho et al. 2007b | EF081120 |
| *P. bocagei* | MOLEDO | 3.36 | Pinho et al. 2007b | EF081120 |
| *P. bocagei* | MOLEDO | 3.37 | Pinho et al. 2007b | EF081120 |
| *P. bocagei* | MOLEDO | 3.40 | this study | KY461815 |
| *P. bocagei* | MOLEDO | 3.41 | Pinho et al. 2007b | EF081120 |
| *P. bocagei* | MOLEDO | 3.43 | Pinho et al. 2007b | EF081120 |
| *P. bocagei* | MOLEDO | 3.45 | Pinho et al. 2007b | EF081131 |
| *P. bocagei* | VPAG | 3.122 | this study | KY461816 |
| *P. bocagei* | VPAG | 3.128 | this study | KY461817 |
| *P. bocagei* | VPAG | 3.129 | this study | KY461818 |
| *P. bocagei* | VPAG | 3.130 | this study | KY461819 |
| *P. bocagei* | VPAG | 3.131 | this study | KY461820 |
| *P. bocagei* | VPAG | 3.132 | this study | KY461821 |
| *P. bocagei* | SMDC | 3.179 | this study | KY461822 |
| *P. bocagei* | SMDC | 3.180 | this study | KY461823 |
| *P. bocagei* | SMDC | 3.182 | this study | KY461824 |
| *P. bocagei* | SMDC | 3.183 | this study | KY461825 |
| *P. bocagei* | SMDC | 3.184 | this study | KY461826 |
| *P. bocagei* | SMDC | 3.186 | this study | KY461827 |
| *P. bocagei* | SMDC | 3.187 | this study | KY461828 |
| *P. bocagei* | SMDC | 3.189 | this study | KY461829 |
| *P. bocagei* | SMDC | 3.203 | this study | KY461830 |
| *P. bocagei* | SMDC | 3.208 | this study | KY461831 |
| *P. bocagei* | SMDC | 3.215 | this study | KY461832 |
| *P. bocagei* | SMDC | 3.216 | this study | KY461833 |
| *P. vaucheri* | BTAZ | DB8775 | Pinho et al. 2007b | EF081108 |
| *P. vaucheri* | BTAZ | DB8776 | Pinho et al. 2007b | EF081107 |
| *P. vaucheri* | BTAZ | DB8777 | Pinho et al. 2007b | EF081108 |
| *P. vaucheri* | BTAZ | DB8778 | Pinho et al. 2007b | EF081108 |
|  |  |  |  |  |
| **Table 5.2.** (continued) | | | | |
| Species | Locality acronym | Sample | Source | Accession number |
| *P. vaucheri* | BTAZ | DB8779 | Pinho et al. 2007b | EF081108 |
| *P. vaucheri* | BTAZ | DB8780/Bt6 | Pinho *et al.* 2006 | DQ081174 |
| *P. vaucheri* | BTAZ | DB8781 | Pinho et al. 2007b | EF081106 |
| *P. vaucheri* | BTAZ | DB8782 | this study | KY461932 |
| *P. vaucheri* | BTAZ | DB9770 | this study | KY461931 |
| *P. vaucheri* | KET | Ket1 | Pinho et al. 2007b | EF081104 |
| *P. vaucheri* | KET | DB8740 | Pinho et al. 2007b | EF081103 |
| *P. vaucheri* | KET | DB8741 | Pinho et al. 2007b | EF081104 |
| *P. vaucheri* | KET | DB8742 | Pinho et al. 2007b | EF081105 |
| *P. vaucheri* | KET | DB8743 | Pinho et al. 2007b | EF081109 |
| *P. vaucheri* | KET | DB8744 | this study | KY461937 |
| *P. vaucheri* | KET | DB8745 | this study | KY461935 |
| *P. vaucheri* | KET | DB8746 | this study | KY461934 |
| *P. vaucheri* | KET | DB8747 | this study | KY461936 |
| *P. vaucheri* | KET | DB8748 | this study | KY461933 |
| *P. vaucheri* | MSCL | DB8804 | Pinho et al. 2007b | EF081096 |
| *P. vaucheri* | MSCL | DB8805 | Pinho et al. 2007b | EF081099 |
| *P. vaucheri* | MSCL | DB8806 | Pinho et al. 2007b | EF081099 |
| *P. vaucheri* | MSCL | DB8807 | Pinho et al. 2007b | EF081091 |
| *P. vaucheri* | MSCL | DB8808 | Pinho et al. 2007b | EF081090 |
| *P. vaucheri* | MSCL | DB8809 | Pinho et al. 2007b | EF081090 |
| *P. vaucheri* | MSCL | DB8810 | Pinho et al. 2007b | EF081094 |
| *P. vaucheri* | MSCL | DB8812 | Pinho et al. 2007b | EF081093 |
| *P. vaucheri* | MSCL | DB8813 | this study | KY461939 |
| *P. vaucheri* | MSCL | DB8814 | this study | KY461940 |
| *P. vaucheri* | MSCL | DB8815 | this study | KY461938 |
| *P. vaucheri* | OUKM | Ouk2 | Pinho et al. 2007b | EF081089 |
| *P. vaucheri* | OUKM | DB8816 | Pinho et al. 2007b | EF081089 |
| *P. vaucheri* | OUKM | DB8817 | Pinho et al. 2007b | EF081089 |
| *P. vaucheri* | OUKM | DB8818 | Pinho et al. 2007b | EF081089 |
| *P. vaucheri* | OUKM | DB8820/Ouk7 | Pinho *et al.* 2006 | DQ081172 |
| *P. vaucheri* | OUKM | DB8822 | Pinho et al. 2007b | EF081089 |
| *P. vaucheri* | OUKM | DB8823 | Pinho et al. 2007b | EF081089 |
| *P. vaucheri* | OUKM | DB8826 | this study | KY461941 |
| *P. vaucheri* | OUKM | DB8827 | this study | KY461942 |
| *P. vaucheri* | OUKM | DB8828 | this study | KY461943 |
| *P. vaucheri* | DEBD | DB8717 | this study | KY461944 |
| *P. vaucheri* | DEBD | DB8718 | this study | KY461945 |
| *P. vaucheri* | DEBD | DB8719 | Pinho et al. 2007b | EF081087 |
| *P. vaucheri* | DEBD | DB8720 | Pinho et al. 2007b | EF081087 |
| *P. vaucheri* | DEBD | DB8721 | this study | KY461946 |
| *P. vaucheri* | DEBD | DB8722 | Pinho et al. 2007b | EF081087 |
| *P. vaucheri* | DEBD | DB8723 | Pinho et al. 2007b | EF081087 |
| **Table 3.2.** (continued) | | | | |
| Species | Locality acronym | Sample | Source | Accession number |
| *P. vaucheri* | DEBD | DB8724 | Pinho et al. 2007b | EF081087 |
| *P. vaucheri* | DEBD | DB8725 | this study | KY461947 |
| *P. vaucheri* | DEBD | DB8726 | Pinho et al. 2007b | EF081087 |
| *P. vaucheri* | TAZA | DB8752 | Pinho et al. 2007b | EF081082 |
| *P. vaucheri* | TAZA | DB8753 | Pinho et al. 2007b | EF081080 |
| *P. vaucheri* | TAZA | DB8754 | Pinho et al. 2007b | EF081086 |
| *P. vaucheri* | TAZA | DB8755 | Pinho et al. 2007b | EF081085 |
| *P. vaucheri* | TAZA | DB8756 | Pinho et al. 2007b | EF081081 |
| *P. vaucheri* | TAZA | DB8757 | this study | KY461950 |
| *P. vaucheri* | TAZA | DB8758 | this study | KY461949 |
| *P. vaucheri* | TAZA | DB8759 | Pinho et al. 2007b | EF081083 |
| *P. vaucheri* | TAZA | DB8760 | Pinho et al. 2007b | EF081080 |
| *P. vaucheri* | TAZA | DB8761 | this study | KY461948 |
| *P. vaucheri* | MDLT | Mid49 | Pinho et al. 2007b | EF081088 |
| *P. vaucheri* | MDLT | Mid50 | Pinho et al. 2007b | EF081088 |
| *P. vaucheri* | MDLT | Mid51 | Pinho et al. 2007b | EF081088 |
| *P. vaucheri* | MDLT | Mid52 | Pinho et al. 2007b | EF081088 |
| *P. vaucheri* | MDLT | Mid53 | Pinho et al. 2007b | EF081088 |
| *P. vaucheri* | MDLT | 7.284 | this study | KY461952 |
| *P. vaucheri* | MDLT | 7.285 | this study | KY461951 |
| *P. vaucheri* | MDLT | 7.286 | this study | KY461953 |
| *P. vaucheri* | MDLT | 7.287 | this study | KY461954 |
| *P. vaucheri* | MDLT | 7.288 | this study | KY461955 |
| *P. vaucheri* | IML | 7.244 | this study | KY461956 |
| *P. vaucheri* | IML | 7.245 | this study | KY461958 |
| *P. vaucheri* | IML | 7.246 | this study | KY461962 |
| *P. vaucheri* | IML | 7.247 | this study | KY461957 |
| *P. vaucheri* | IML | 7.248 | this study | KY461963 |
| *P. vaucheri* | IML | 7.249 | this study | KY461959 |
| *P. vaucheri* | IML | 7.250 | this study | KY461960 |
| *P. vaucheri* | IML | 7.251 | this study | KY461961 |
| *P. vaucheri* | IML | 7.252 | Kaliontzopoulou *et al.* 2011 | HQ898028 |
| *P. vaucheri* | IML | 7.260 | this study | KY461964 |
| *P. vaucheri* | TISL | 7.377 | Kaliontzopoulou *et al*. 2011 | HQ898032 |
| *P. vaucheri* | TISL | 7.396 | this study | KY461965 |
| *P. vaucheri* | TISL | 7.399 | this study | KY461966 |
| *P. vaucheri* | TISL | 7.400 | this study | KY461967 |
| *P. vaucheri* | TISL | 7.401 | this study | KY461969 |
| *P. vaucheri* | TISL | 7.403 | this study | KY461968 |
| *P. vaucheri* | TISL | 7.404 | this study | KY461970 |
| *P. vaucheri* | TISL | 7.405 | this study | KY461971 |
| *P. vaucheri* | TISL | 7.482 | this study | KY461972 |
|  |  |  |  |  |

**5.1.2.2 Analytical procedures**

Alignment was carried out manually against published datasets using Bioedit 7.2.5 (Hall *et al.* 1999). Both the *P. bocagei* and the *P. vaucheri* alignments were trimmed to a common portion of 623bp, covering a portion of the ND4 gene incomplete at both the 5’ and 3’ ends.

Using these trimmed alignments, we calculated diversity measures per locality (haplotype diversity, Hd and nucleotide diversity, π) taking into account differences between sample sizes using the same exact procedure detailed in Salvi *et al.* 2013 and outlined above for microsatellite data. This procedure implies generating sets of resampled sequences per population with the minimal sample size observed across populations and analyzing these sets using the “batch mode” option in DNAsp (Librado and Rozas 2009). The mean of these measures per population was then taken. Also using DNAsp, we calculated Dxy, the average number of nucleotide substitutions per site between populations (Nei 1987). Finally, we calculated Hudson, Slatkin and Maddison (1992) Fst between populations. Because DNAsp does not implement a permutation test to evaluate the significance of this measure, we used Python scripts to accomplish this task. Python scripts were also used for all format conversions, resampling of datasets and complex calculations. All scripts are available from the authors upon request.

**5.2 Results**

**5.2.1 Microsatellites**

The number of alleles per locus was considerably high (ranging from 23 alleles in *Pb11* and *Pb20* to 55 in *Pb10* in *P. bocagei* and from 22 in *Pb11* to 62 in *Pb55* in *P. vaucheri*), as previously reported for these markers (Pinho *et al.* 2004, 2011).

Analyses using Microchecker revealed the presence of null alleles in most of the loci in at least one population per species (results not shown), with an extreme in *Pb11, Pb37* and *Pb47* in *P. vaucheri* (in which 8, 7 and 7, respectively, out of 9 populations had evidence for the presence of null alleles) and in *Pb20* in *P. bocagei* (in 7 out of 9 populations). The prevalence of this type of genotyping problem across loci was the reason to use corrections for null alleles using FreeNa (see 5.1.1.2).

Diversity measures are presented in table 5.3 for both species.

| \| **Table 5.3.** Diversity in *P. bocagei* and *P. vaucheri* populations based on 9 microsatellite loci \| \| \| \| \| \| \| \| \| \| --- \| --- \| --- \| --- \| --- \| --- \| --- \| --- \| --- \| \| *P. bocagei* \| \| \| \|  \| *P. vaucheri* \| \| \| \| \|  \| Avg(N) \| Na \| He \|  \|  \| Avg(N) \| Na \| He \| \| CLAB \| 28.56 \| 16.56 \| 0.8852 \|  \| BTAZ \| 34.33 \| 15.99 \| 0.8422 \| \| GERES \| 33.33 \| 15.73 \| 0.8939 \|  \| DEBD \| 21.00 \| 11.85 \| 0.8121 \| \| GIAO \| 32.56 \| 12.67 \| 0.8706 \|  \| IML \| 20.89 \| 13.09 \| 0.7991 \| \| MDLN \| 33.44 \| 12.89 \| 0.8665 \|  \| KET \| 21.56 \| 15.15 \| 0.8323 \| \| MNTS \| 30.44 \| 15.52 \| 0.885 \|  \| MDLT \| 24.56 \| 10.78 \| 0.7746 \| \| MOLEDO \| 26.33 \| 14.68 \| 0.8621 \|  \| MSCL \| 20.56 \| 13.55 \| 0.837 \| \| SMDC \| 32.44 \| 14.79 \| 0.8735 \|  \| OUKM \| 24.78 \| 12.00 \| 0.8216 \| \| SUBPOR \| 34.44 \| 14.37 \| 0.8655 \|  \| TAZA \| 30.33 \| 16.54 \| 0.8542 \| \| VPAG \| 32.78 \| 12.56 \| 0.8534 \|  \| TISL \| 31.78 \| 13.01 \| 0.8434 \| |  |  |  |
| --- | --- | --- | --- | --- | --- | --- | --- | --- | --- | --- | --- | --- | --- | --- | --- | --- | --- | --- | --- | --- | --- | --- | --- | --- | --- | --- | --- | --- | --- | --- | --- | --- | --- | --- | --- | --- | --- | --- | --- | --- | --- | --- | --- | --- | --- | --- | --- | --- | --- | --- | --- | --- | --- | --- | --- | --- | --- | --- | --- | --- | --- | --- | --- | --- | --- | --- | --- | --- | --- | --- | --- | --- | --- | --- | --- | --- | --- | --- | --- | --- | --- | --- | --- | --- | --- | --- | --- | --- | --- | --- | --- | --- | --- | --- | --- | --- | --- | --- | --- | --- | --- | --- | --- | --- | --- | --- | --- | --- | --- | --- | --- |

Notes: Avg(N): mean sample size across loci; Na: mean number of alleles per locus corrected for differential sample size; He: expected heterozygosity based on allele frequencies corrected for null alleles. See table Supplementary Table 1 for locality acronyms.

Overall Fst across loci based on the ENA correction implemented in FreeNa was roughly similar among species: 0.0321 for *P. bocagei* and 0.0320 for *P. vaucheri*, both of them significant (p<0.01) based on 100 permutations*.* Pairwise Fst between populations are shown in tables 5.4 and 5.5 for *P. bocagei* and *P. vaucheri*, respectively.

| **Table 5.4** Pairwise Fst between *P. bocagei* populations corrected for null alleles according to the ENA correction (Chapuis & Estoup 2007). See table Supplementary Table 1 for locality acronyms. | | | | | | | | |
| --- | --- | --- | --- | --- | --- | --- | --- | --- |
|  | CLAB | GERES | GIAO | MDLN | MNTS | MOLEDO | SMDC | SUBPOR |
| GERES | 0.01030 |  |  |  |  |  |  |  |
| GIAO | 0.02147 | 0.02689 |  |  |  |  |  |  |
| MDLN | 0.03932 | 0.04580 | 0.03822 |  |  |  |  |  |
| MNTS | 0.02410 | 0.01794 | 0.02808 | 0.04128 |  |  |  |  |
| MOLEDO | 0.02997 | 0.03243 | 0.03876 | 0.04939 | 0.04497 |  |  |  |
| SMDC | 0.02605 | 0.02506 | 0.02534 | 0.04384 | 0.01374 | 0.03928 |  |  |
| SUBPOR | 0.01605 | 0.01807 | 0.02705 | 0.05334 | 0.03264 | 0.02865 | 0.02620 |  |
| VPAG | 0.03189 | 0.03079 | 0.03679 | 0.04447 | 0.02545 | 0.05619 | 0.02160 | 0.04547 |

Note: all values are significant (p<0.01) based on 100 permutations.

| **Table 5.5.** Pairwise Fst between *P. vaucheri* populations corrected for null alleles according to the ENA correction (Chapuis & Estoup 2007). See table Supplementary Table 1 for locality acronyms. | | | | | | | | |
| --- | --- | --- | --- | --- | --- | --- | --- | --- |
|  | BTAZ | DEBD | IML | KET | MDLT | MSCL | OUKM | TAZA |
| DEBD | 0.03419 |  |  |  |  |  |  |  |
| IML | 0.03345 | 0.04741 |  |  |  |  |  |  |
| KET | 0.00870 | 0.02528 | 0.02795 |  |  |  |  |  |
| MDLT | 0.03241 | 0.04585 | 0.04435 | 0.03331 |  |  |  |  |
| MSCL | 0.02687 | 0.04603 | 0.02805 | 0.02198 | 0.03476 |  |  |  |
| OUKM | 0.03474 | 0.05443 | 0.02406 | 0.03402 | 0.05133 | 0.03555 |  |  |
| TAZA | 0.01087 | 0.03005 | 0.02740 | 0.00691 | 0.03087 | 0.02149 | 0.03331 |  |
| TISL | 0.03091 | 0.04814 | 0.03068 | 0.02768 | 0.04433 | 0.01766 | 0.03220 | 0.02612 |

Note: all values are significant (p<0.01) except for TAZA vs DEBD (0.01<p<0.05) based on 100 permutations.

Cavalli-Sforza and Edwards (1967) chord distances between populations, calculated using the INA correction for null alleles, are presented in tables 5.6 and 5.7 for *P. bocagei* and *P. vaucheri*, respectively.

| **Table 5.6.** Cavalli-Sforza & Edwards (1967) chord distance between *P. bocagei* populations corrected for null alleles according to the INA correction (Chapuis & Estoup 2007). See table Supplementary Table 1 for locality acronyms. | | | | | | | | |
| --- | --- | --- | --- | --- | --- | --- | --- | --- |
|  | CLAB | GERES | GIAO | MDLN | MNTS | MOLEDO | SMDC | SUBPOR |
| GERES | 0.40685 |  |  |  |  |  |  |  |
| GIAO | 0.46525 | 0.46494 |  |  |  |  |  |  |
| MDLN | 0.50307 | 0.51343 | 0.47828 |  |  |  |  |  |
| MNTS | 0.47002 | 0.43365 | 0.45753 | 0.50903 |  |  |  |  |
| MOLEDO | 0.48795 | 0.45301 | 0.52467 | 0.56748 | 0.53625 |  |  |  |
| SMDC | 0.45782 | 0.44197 | 0.40300 | 0.46994 | 0.40489 | 0.52759 |  |  |
| SUBPOR | 0.39946 | 0.41142 | 0.45710 | 0.51650 | 0.46941 | 0.42841 | 0.44931 |  |
| VPAG | 0.48329 | 0.47002 | 0.45696 | 0.49133 | 0.44504 | 0.55884 | 0.38691 | 0.49057 |

| **Table 5.7.** Cavalli-Sforza & Edwards (1967) chord distances between *P. vaucheri* populations corrected for null alleles according to the INA correction (Chapuis & Estoup 2007). See table Supplementary Table 1 for locality acronyms. | | | | | | | | |
| --- | --- | --- | --- | --- | --- | --- | --- | --- |
|  | BTAZ | DEBD | IML | KET | MDLT | MSCL | OUKM | TAZA |
| DEBD | 0.52610 |  |  |  |  |  |  |  |
| IML | 0.51888 | 0.56084 |  |  |  |  |  |  |
| KET | 0.43474 | 0.48329 | 0.53123 |  |  |  |  |  |
| MDLT | 0.50460 | 0.53459 | 0.53505 | 0.52657 |  |  |  |  |
| MSCL | 0.51021 | 0.57298 | 0.49202 | 0.53055 | 0.50026 |  |  |  |
| OUKM | 0.52845 | 0.57857 | 0.44554 | 0.56395 | 0.55101 | 0.50251 |  |  |
| TAZA | 0.42862 | 0.49608 | 0.48706 | 0.46671 | 0.51471 | 0.51910 | 0.51740 |  |
| TISL | 0.50555 | 0.54145 | 0.48559 | 0.52049 | 0.51808 | 0.45231 | 0.48545 | 0.49483 |

**5.2.2 Mitochondrial DNA**

We obtained new sequence data for 57 *P. bocagei* and 42 *P. vaucheri* individuals. In total, combined with previously published data, our data set included 81 and 89 *P. bocagei* and *P. vaucheri* sequences, respectively.

Diversity measures for the total datasets and per population are shown in table 5.8 and 5.9 for *P. bocagei* and *P. vaucheri*, respectively. Note that these numbers cannot be directly compared to those presented in previous papers (namely Pinho et al. 2007b) because of the shorter alignment size used in the present study. However, the results of much higher diversity levels in *P. vaucheri* when compared to *P. bocagei* conform in total to previously published data.

Global Fst was 0.49 for *P. bocagei* and 0.88 for *P. vaucheri* and both values were significant (p<0.001). Pairwise Fst between populations are shown in tables 5.10 and 5.11 for *P. bocagei* and *P. vaucheri*, respectively.

Dxy, calculated according to Nei (1987), is shown on table 5.12 and 5.13 in *P. bocagei* and *P. vaucheri*, respectively.

| **Table 5.8** Diversity measures in *P. bocagei* populations. | | | | | |
| --- | --- | --- | --- | --- | --- |
| Group | n | S | Hap | Hd | π |
| Total dataset | 81 | 9 | 10 | 0.600 | 0.00189 |
| CLAB observed | 7 | 1 | 2 | 0.286 | 0.00060 |
| CLAB resampled | 6 | 0.86 | 1.86 | 0.286 | 0.00060 |
| GERES observed | 7 | 2 | 3 | 0.667 | 0.00159 |
| GERES resampled | 6 | 1.81 | 2.81 | 0.663 | 0.00158 |
| GIAO observed | 8 | 0 | 1 | 0.000 | 0.00000 |
| GIAO resampled | 6 | 0 | 1 | 0.000 | 0.00000 |
| MDLN observed | 12 | 1 | 2 | 0.409 | 0.00085 |
| MDLN resampled | 6 | 0.88 | 1.88 | 0.393 | 0.00082 |
| MNTS observed | 12 | 1 | 2 | 0.409 | 0.00085 |
| MNTS resampled | 6 | 0.9 | 1.9 | 0.414 | 0.00087 |
| MOLEDO observed | 9 | 3 | 3 | 0.556 | 0.00255 |
| MOLEDO resampled | 6 | 2.63 | 2.59 | 0.569 | 0.00258 |
| SMDC observed | 12 | 3 | 4 | 0.455 | 0.00104 |
| SMDC resampled | 6 | 1.55 | 2.55 | 0.471 | 0.00108 |
| SUBPOR observed | 8 | 1 | 2 | 0.429 | 0.00090 |
| SUBPOR resampled | 6 | 0.97 | 1.97 | 0.419 | 0.00088 |
| VPAG observed | 6 | 0 | 1 | 0.000 | 0.00000 |
| NOTE: resampled values calculated according to Salvi *et al.* 2013 based on a sample size of 6 individuals. n, number of sequences; S, number of segregating sites; Hap, number of haplotypes; Hd, haplotype diversity; π, nucleotide diversity | | | | | |

| **Table 5.9** Diversity measures in *P. vaucheri* populations. | | | | | |
| --- | --- | --- | --- | --- | --- |
| Group | n | S | Hap | Hd | π |
| Total dataset | 89 | 69 | 33 | 0.946 | 0.02771 |
| BTAZ observed | 9 | 2 | 3 | 0.667 | 0.01097 |
| DEBD observed | 10 | 0 | 1 | 0.000 | 0.00000 |
| DEBD resampled | 9 | 0 | 1 | 0.000 | 0.00000 |
| IML observed | 10 | 12 | 3 | 0.689 | 0.00901 |
| IML resampled | 9 | 12 | 3 | 0.695 | 0.00911 |
| KET observed | 10 | 12 | 6 | 0.889 | 0.00682 |
| KET resampled | 9 | 11.81 | 5.69 | 0.890 | 0.00686 |
| MDLT observed | 10 | 0 | 1 | 0.000 | 0.00000 |
| MDLT resampled | 9 | 0 | 1 | 0.000 | 0.00000 |
| MSCL observed | 11 | 9 | 6 | 0.855 | 0.00621 |
| MSCL resampled | 9 | 8.66 | 5.47 | 0.859 | 0.00620 |
| OUKM observed | 10 | 0 | 1 | 0.000 | 0.00000 |
| OUKM resampled | 9 | 0 | 1 | 0.000 | 0.00000 |
| TAZA observed | 10 | 12 | 8 | 0.956 | 0.00551 |
| TAZA resampled | 9 | 11.03 | 7.49 | 0.958 | 0.00543 |
| TISL observed | 9 | 6 | 6 | 0.917 | 0.00526 |
| NOTE: resampled values calculated according to Salvi *et al.* 2013 based on a sample size of 9 individuals. n, number of sequences; S, number of segregating sites; Hap, number of haplotypes; Hd, haplotype diversity; π, nucleotide diversity | | | | | |

| **Table 5.10.** Pairwise Fst between *P. bocagei* populations calculated according to Hudson *et al.* (1992) | | | | | | | | |
| --- | --- | --- | --- | --- | --- | --- | --- | --- |
|  | CLAB | GERES | GIAO | MDLN | MNTS | MOLEDO | SMDC | SUBPOR |
| GERES | 0.083 |  |  |  |  |  |  |  |
| GIAO | 0.000 | 0.111 |  |  |  |  |  |  |
| MDLN | 0.116 | 0.137 | 0.182 |  |  |  |  |  |
| MNTS | **0.751** | **0.651** | **0.836** | **0.727** |  |  |  |  |
| MOLEDO | **0.525** | **0.470** | **0.577** | **0.519** | **0.655** |  |  |  |
| SMDC | 0.000 | 0.036 | 0.000 | 0.091 | **0.659** | **0.492** |  |  |
| SUBPOR | -0.111 | 0.123 | 0.143 | 0.162 | **0.721** | **0.513** | **0.071** |  |
| VPAG | 0.000 | 0.111 | 0.000 | 0.182 | **0.836** | **0.577** | 0.000 | 0.143 |
| Note: significant values (p<0.05) are shown in bold | | | | |  |  |  |  |

| **Table 5.11.** Pairwise Fst between *P. vaucheri* populations calculated according to Hudson *et al.* (1992) | | | | | | | | | |
| --- | --- | --- | --- | --- | --- | --- | --- | --- | --- |
|  | BTAZ | DEBD | KET | IML | MDLT | MSCL | OUKM | TAZA | TISL |
| BTAZ |  |  |  |  |  |  |  |  |  |
| DEBD | 0.985 |  |  |  |  |  |  |  |  |
| KET | 0.221 | 0.912 |  |  |  |  |  |  |  |
| IML | 0.878 | 0.853 | 0.799 |  |  |  |  |  |  |
| MDLT | 0.986 | 1.000 | 0.922 | 0.869 |  |  |  |  |  |
| MSCL | 0.910 | 0.889 | 0.831 | 0.591 | 0.903 |  |  |  |  |
| OUKM | 0.981 | 1.000 | 0.890 | 0.653 | 1.000 | 0.783 |  |  |  |
| TAZA | 0.903 | 0.866 | 0.817 | 0.772 | 0.904 | 0.797 | 0.900 |  |  |
| TISL | 0.941 | 0.944 | 0.864 | 0.696 | 0.946 | 0.756 | 0.872 | 0.868 |  |
| Note: all values are significant (p<0.05) | | | | | | | | | |

| **Table 5.12.** Dxy between *P. bocagei* populations calculated according to Nei (1987) | | | | | | | | | |
| --- | --- | --- | --- | --- | --- | --- | --- | --- | --- |
|  | CLAB | GERES | GIAO | MDLN | MNTS | MOLEDO | SMDC | SUBPOR | VPAG |
| CLAB |  |  |  |  |  |  |  |  |  |
| GERES | 0.001 |  |  |  |  |  |  |  |  |
| GIAO | 0.000 | 0.001 |  |  |  |  |  |  |  |
| MDLN | 0.001 | 0.001 | 0.001 |  |  |  |  |  |  |
| MNTS | 0.003 | 0.004 | 0.003 | 0.003 |  |  |  |  |  |
| MOLEDO | 0.003 | 0.004 | 0.003 | 0.004 | 0.005 |  |  |  |  |
| SMDC | 0.001 | 0.001 | 0.001 | 0.001 | 0.003 | 0.004 |  |  |  |
| SUBPOR | 0.001 | 0.001 | 0.001 | 0.001 | 0.003 | 0.004 | 0.001 |  |  |
| VPAG | 0.000 | 0.001 | 0.000 | 0.001 | 0.003 | 0.003 | 0.001 | 0.001 |  |

| **Table 5.13.** Dxy between *P. vaucheri* populations calculated according to Nei (1987) | | | | | | | | | |
| --- | --- | --- | --- | --- | --- | --- | --- | --- | --- |
|  | BTAZ | DEBD | KET | IML | MDLT | MSCL | OUKM | TAZA | TISL |
| BTAZ |  |  |  |  |  |  |  |  |  |
| DEBD | 0.042 |  |  |  |  |  |  |  |  |
| KET | 0.005 | 0.039 |  |  |  |  |  |  |  |
| IML | 0.042 | 0.031 | 0.039 |  |  |  |  |  |  |
| MDLT | 0.047 | 0.026 | 0.044 | 0.034 |  |  |  |  |  |
| MSCL | 0.042 | 0.028 | 0.039 | 0.019 | 0.032 |  |  |  |  |
| OUKM | 0.034 | 0.025 | 0.031 | 0.013 | 0.033 | 0.014 |  |  |  |
| TAZA | 0.035 | 0.021 | 0.034 | 0.032 | 0.029 | 0.029 | 0.027 |  |  |
| TISL | 0.042 | 0.032 | 0.038 | 0.021 | 0.034 | 0.020 | 0.014 | 0.035 |  |

**References not cited in the main text**

Arévalo E, Davis SK, Sites Jr. JW (1994) Mitochondrial DNA sequence divergence and phylogenetic relationships among eight chromosome races of the *Sceloporus grammicus* complex (Phrynosomatidae) in central Mexico. *Systematic Biology* **43:**387–418.

Chapuis MP, Estoup A (2007). Microsatellite null alleles and estimation of population differentiation. *Molecular biology and evolution* **24**: 621-631.

Hall TA (1999) BioEdit: a user-friendly biological sequence alignment editor and analysis program for Windows 95/98/NT. *Nucleic Acids Symp Ser*  **41:** 95–98

Librado P, Rozas J (2009) DnaSP v5: A software for comprehensive analysis of DNA polymorphism data. *Bioinformatics* **25**: 1451-1452

Oetting WS, Lee HK, Flanders DJ, Wiesner GL, Sellers TA, King RA (1995). Linkage analysis with multiplexed short tandem repeat polymorphisms using infrared fluorescence and M13 tailed primers. *Genomics* **30:**450-458.

Pinho C, Ferrand N, Harris DJ (2006) Reexamination of the Iberian and North African *Podarcis* (Squamata: Lacertidae) phylogeny based on increased mitochondrial DNA sequencing. *Molecular Phylogenetics and Evolution* **38:** 266-273

Salvi D, Harris DJ, Kaliontzopoulou A, Carretero MA, Pinho C (2013) Persistence across Pleistocene Ice Ages in Mediterranean and extra-Mediterranean refugia: phylogeographic insights from the common wall lizard. *BMC Evolutionary Biology* **13:**147.

Sambrook J, Fritsch EF, Maniatis T (1989) Molecular Cloning: a Laboratory Manual, 2^nd^ edn. Cold Spring Harbor Press, New York

Van Oosterhout C, Hutchinson WF, Wills DP, Shipley P(2004). MICRO‐CHECKER: software for identifying and correcting genotyping errors in microsatellite data. *Molecular Ecology Notes*, **4**: 535-538
