# Supplementary material for: Where does diversity come from? Linking geographical patterns of morphological, genetic, and environmental variation in wall lizards
Source: BMC Evol Biol. 2018 Aug 22;18:124. doi: 10.1186/s12862-018-1237-7 (PMC6113677; doi:10.1186/s12862-018-1237-7)
Supplement: Supplementary file 9 — Maxent models for quantifying environmental suitability, provides a description of methods and results related to ENM used to obtain habitat suitability maps for each species. (DOCX 727 kb) [file 12862_2018_1237_MOESM9_ESM.docx]

**Additional file 7:** Maxent models for quantifying environmental suitability

**7.1. Methods**

Species environmental suitability was calculated using the Maximum Entropy ecological modelling approach in the Maxent ver. 3.3.k software (Phillips et al., 2006), which requires presence-only records and performs well in comparison to other methods (Elith et al., 2006, 2011). We used 38 and 50 presence records for *P. bocagei* and *P. vaucheri*, respectively, coming from already genetically analysed specimens along the whole distributional range of each species (Kaliontzopoulou *et al*., 2011; Caeiro-Dias *et al*., in press). As predictors, we used seven slightly correlated variables (R<0.7), including six climatic and slope, at 30 arc seconds of resolution, also used in other analyses (see main text). The six climatic variables (i.e. BIO3, BIO5, BIO7, BIO8, BIO13 and BIO14) were downloaded from WorldClim Portal (http://www.worldclim.org/). The slope variable was derived from altitude (available also at WorldClim) and calculated using the “slope” function of ArcGis (ESRI, 2014). In other to properly delimit study areas for modelling purposes (see Anderson & Raza, 2010), we cut variables by a 150 km buffer around minimum convex polygons including all presence records for each species.

In Maxent and for each species, 20 model replicates were run with random seed and 80% training/20% testing data partition in each run. Samples for each replicate were chosen by bootstrap, allowing sampling with replacement. Models were run with auto-features (Phillips *et al*., 2006), and the area under the curve (AUC) of the receiver-operating characteristics (ROC) plots was taken as a measure of individual model fit (Fielding & Bell, 1997). The individual model replicates were added to generate an average model of environmental suitability (Marmion *et al*., 2009). Standard deviation between individual model replicates was used as an indication of prediction uncertainty.

**7.2. Results**

In both species, average values for training and test AUC were high, while standard deviations were low (Table A7), suggesting a good performance for models. The model obtained for *P. bocagei* predicted higher suitability for populations near to the Atlantic Ocean than for those located inland (Fig. A7). The model obtained for *P. vaucheri* restricted the areas with high suitability to most of the North African mountain ranges occupied by the species (Fig. A7).

**Table A7.** Number of training and test samples, training and testing AUC, and average (and standard deviation) contribution of variables to each species model.

| **Metrics - Variables** | ***P. bocagei*** | ***P. vaucheri*** |
| --- | --- | --- |
| trainning / test samples | 31 / 7 | 40 / 9 |
| Training AUC | 0.88 (0.026) | 0.934 (0.011) |
| Test AUC | 0.806 (0.09) | 0.905 (0.07) |
| BIO3 | 16.27 (8.9) | 5.82 (3.54) |
| BIO5 | 7.73 (7.95) | 3.8 (2.71) |
| BIO7 | 37.05 (12.9) | 10.32 (5.9) |
| BIO8 | 2.83 (2.9) | 17.15 (11.26) |
| BIO13 | 12.6 (10.09) | 32.2 (7.8) |
| BIO15 | 15.22 (8.87) | 10.23 (5.93) |
| Slope | 8.29 (6.3) | 20.5 (10.6) |

**Figure A7.** Environmental suitability for *P. bocagei* (left) and *P. vaucheri* (right) derived with Maxent.


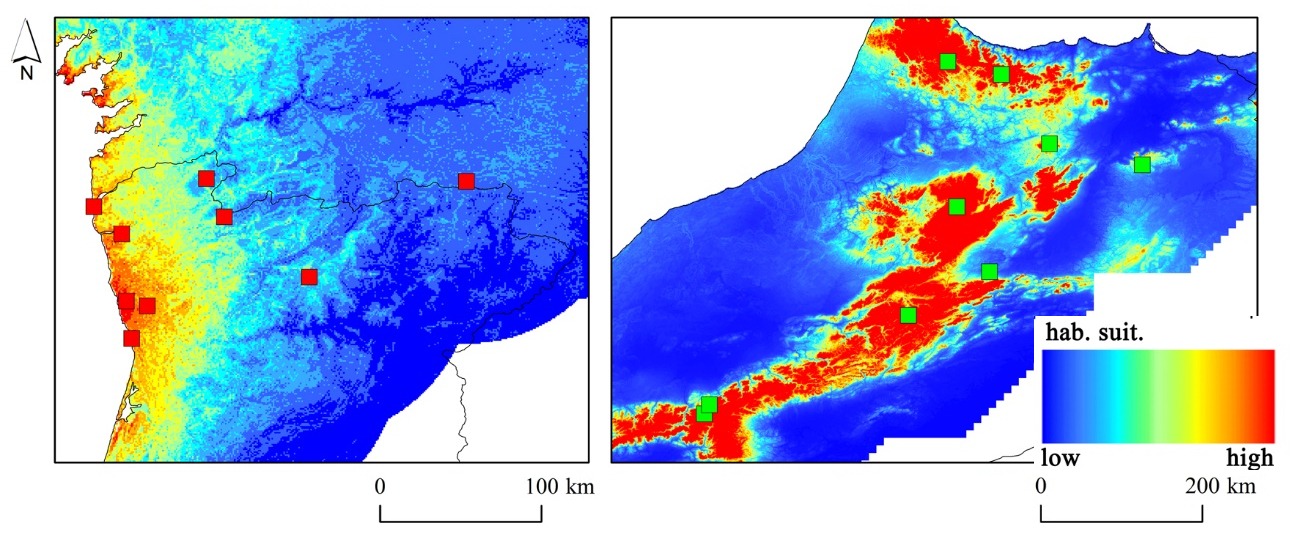


**References not included in the main text**

Anderson, R.P. & Raza, A. (2010) The effect of the extent of the study region on GIS models of species geographic distributions and estimates of niche evolution: preliminary tests with montane rodents (genus *Nephelomys*) in Venezuela. *Journal of Biogeography*, 37, 1378–1393.

Elith J., H. Graham C., P. Anderson R., Dudík M., Ferrier S., Guisan A., J. Hijmans R., Huettmann F., R. Leathwick J., Lehmann A., Li J., G. Lohmann L., A. Loiselle B., Manion G., Moritz C., Nakamura M., Nakazawa Y., McC. M. Overton J., Townsend Peterson A., J. Phillips S., Richardson K., Scachetti-Pereira R., E. Schapire R., Soberón J., Williams S., S. Wisz M., & E. Zimmermann N. (2006) Novel methods improve prediction of species’ distributions from occurrence data. *Ecography*, 29, 129–151.

Elith, J., Phillips, S. J., Hastie, T., Dudík, M., Chee, Y. E., & Yates, C. J. (2011). A statistical explanation of MaxEnt for ecologists. *Diversity and distributions*, 17(1), 43-57.

Fielding A.H. & Bell J.F. (1997) A review of methods for the assessment of prediction errors in conservation presence/absence models. *Environmental Conservation*, 24, 38–49.

Marmion M., Parviainen M., Luoto M., Heikkinen R.K., & Thuiller W. (2009) Evaluation of consensus methods in predictive species distribution modelling. *Diversity and Distributions*, 15, 59–69.
